# Material Engineering in Gut Microbiome and Human Health

**DOI:** 10.34133/2022/9804014

**Published:** 2022-07-20

**Authors:** Letao Yang, Lin Y. Hung, Yuefei Zhu, Suwan Ding, Kara G. Margolis, Kam W. Leong

**Affiliations:** ^1^Department of Biomedical Engineering, Columbia University, New York, NY, USA; ^2^Department of Pediatrics, Columbia University, New York, New York, USA; ^3^Department of Systems Biology, Columbia University, New York, NY, USA

## Abstract

Tremendous progress has been made in the past decade regarding our understanding of the gut microbiome's role in human health. Currently, however, a comprehensive and focused review marrying the two distinct fields of gut microbiome and material research is lacking. To bridge the gap, the current paper discusses critical aspects of the rapidly emerging research topic of “material engineering in the gut microbiome and human health.” By engaging scientists with diverse backgrounds in biomaterials, gut-microbiome axis, neuroscience, synthetic biology, tissue engineering, and biosensing in a dialogue, our goal is to accelerate the development of research tools for gut microbiome research and the development of therapeutics that target the gut microbiome. For this purpose, state-of-the-art knowledge is presented here on biomaterial technologies that facilitate the study, analysis, and manipulation of the gut microbiome, including intestinal organoids, gut-on-chip models, hydrogels for spatial mapping of gut microbiome compositions, microbiome biosensors, and oral bacteria delivery systems. In addition, a discussion is provided regarding the microbiome-gut-brain axis and the critical roles that biomaterials can play to investigate and regulate the axis. Lastly, perspectives are provided regarding future directions on how to develop and use novel biomaterials in gut microbiome research, as well as essential regulatory rules in clinical translation. In this way, we hope to inspire research into future biomaterial technologies to advance gut microbiome research and gut microbiome-based theragnostics.

## 1. Introduction

After Louis Pasteur raised the seminal idea that bacteria are a major mediator for many infectious diseases, it took nearly two additional centuries before investigators revealed that the microbiome in fact plays many complex and multifaceted roles in human health and disease [1, 2]. For instance, animals that are germ-free or treated with broad-spectrum antibiotics exhibit significantly altered metabolic levels, trophic factors, enzymatic activities, and specific lipids [3]. Further, microbial dysbiosis has been associated with neural, intestinal, cardiac, renal, and immune disorders [4].

This better understanding of the gut microbiome has already resulted in novel therapeutic strategies and clinical trials [5, 6]. Initial phases of the Human Microbiome Project and MetaHIT8 have also highlighted the importance and future directions of the gut microbiome in human health and diseases [7]. Specifically, advanced multiomic tools including metagenomics, metametabolomics, metaproteomics, and metatranscriptomics have suggested that host-microbiome interactions are significantly more dynamic than what we knew a decade ago [8, 9]. For instance, in addition to taxonomic composition, molecular functions of prevalent microbial species and personalized, strain-specific populations may play important roles in the symbiosis and health status of hosts [10–12].

Expanding the capacity of multiomic approaches is necessary to pave the road for microbiome research over the next decade on microbial pathways, virome and fungome studies, and their dynamic interactions with host protein and metabolite-driven pathways [13]. Innovative models that enable these multiomic studies on the human microbiome and effective strategies for regulating the dynamic and diverse microbiome are frontiers of the next decade of gut microbiome research [7]. Better characterization, a deeper understanding, and advanced therapeutic approaches may significantly impact the clinical treatment of many human diseases.

Materials have played vital roles in nearly all aspects of microbial research [14]. Small molecules or biomolecules, such as antibiotics, have been used in clinics for almost a century. More recently, the development of metagenomic-, metametabolomic-, metaproteomic-, and metatranscriptomic-based technologies has involved material engineering, including surface patterning techniques, fluorescent bead labeling techniques, microfluidic devices, and optical sensing technologies [15–17].

Breakthroughs in the *in vitro* modeling of human gut microbiomes have also been built upon intestinal organoids coupled with physiologically relevant soft materials (e.g., hydrogels) or gut-on-chip devices [18]. The interplay between gut microbiota and natural and synthetic materials is even more profoundly reshaping microbiome research [19]. For instance, mucin in the gut mucosal layer is constantly regulated and simultaneously remodeled by the surrounding microbiota that not only directly alters the dynamics of metabolism in the gut but also influences the efficacies of many drugs that can eventually impact disease treatment [20]. Meanwhile, oral delivery devices have successfully delivered microbiota for the regulation of the gut microbiome [21].

Biomaterials show enormous potential in facilitating the understanding, modeling, and manipulating of the gut microbiome. Establishing a framework for material-facilitated gut microbiome research could thus significantly accelerate the clinical translation of this new direction for broad biomedical applications.

**Figure 1:**
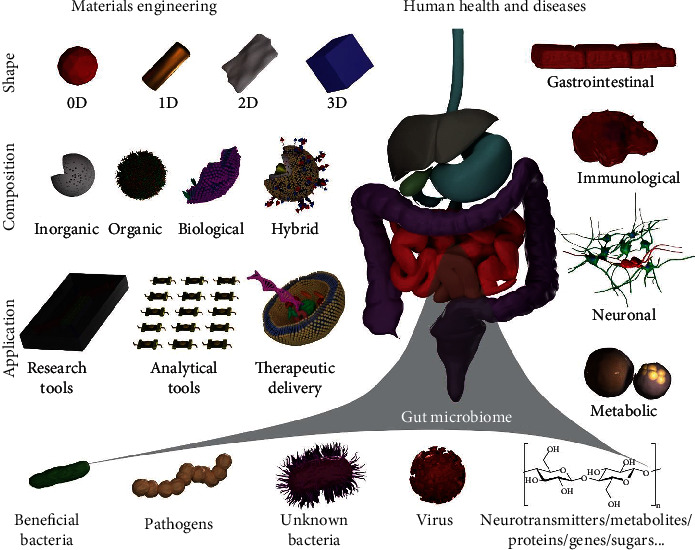
Material engineering in gut microbiome and human health. Materials can be engineered in terms of size, structure, and composition to achieve different functions for the study, modeling, analysis, and manipulation of the gut microbiome and can eventually facilitate treatment of gastrointestinal, immunological, neuronal, and metabolic diseases and cancer, thereby benefiting human health.

This current review (Figure 1) describes contemporary research and understanding of the gut microbiome, the gut-brain axis, and their roles in various types of diseases. Subsequently, a summary is provided regarding the current development of material-based engineering approaches for understanding, modeling, and manipulating the gut microbiome and associated diseases. Lastly, challenges and opportunities are highlighted in the development of novel materials for microbiome research.

Collaborations among gastroenterologists, biologists, material scientists, bioengineers, and clinicians of other disciplines will further advance an understanding of gut microbiome-related diseases and facilitate new therapeutic strategies enabled by material engineering. Robust platform technologies built upon advanced biomaterials will thus facilitate and contribute to emerging gut microbiome research.

## 2. Gut Microbiome

The gut microbiome is composed of bacteria, viruses, archaea, protists, and fungi, with a total cell number even higher (by around 12%) than the host itself [22]. These microorganisms typically inhabit the small intestine and colon in the lower GI tract, where they perform a dynamic and diverse array of metabolic activities [1]. Across the mucosa, biomolecules are under constant flux between the host and the microorganism, which eventually connects the gut microbiota to the whole body.

Such bidirectional communication and host-microbiome interactions are initiated immediately after birth with microbial consortia delivered from the mother [23]. Gut microbiota continues to diversify, followed by a second stage that is relatively resilient to environmental factors (e.g., antibiotics and age) [24]. Due to these developmental differences, the gut microbiota exhibit high variability among different individuals and also at different locations in the GI tract [12]. Greater similarities exist, however, among family members, possibly originating from shared environmental factors (e.g., diet) and/or genetic relatedness [25, 26].

### 2.1. Gut Microbiome and Human Diseases

The gut microbiota has been studied in the context of human development and disease [27, 28]. For example, there is increasing evidence suggesting that host health can be negatively affected by specific microbiota in many cardiovascular, inflammatory, gastrointestinal, metabolic, and neurologic disorders [29]. For instance, some studies have shown that subsets of inflammatory bowel disease (IBD) patients possess a gut microbiota characterized by a decreased population of Bacteroidetes and an increased population of Proteobacteria and phyla Actinobacteria, compared to healthy subjects [30]. Similarly, type 2 diabetic (T2DB) patients often show reduced Firmicutes bacteria while harboring a denser population of Bacteroidetes bacteria [31]. Necrotizing enterocolitis has been associated with higher populations of Proteobacteria and Firmicutes [32]. In contrast, Bacteroidetes and Fusobacteria species are related to healthy infants. Despite these associations between gut microbiota and diseases, it remains unclear whether dysbiosis is a cause or consequence of host health status or other pervasive factors (e.g., diet).

Gain-of-function studies based on microbiota transplantation have begun to provide a more in-depth understanding of the relationship between gut microbiota and host diseases [33]. Nevertheless, such studies have been challenging and are in their infancy. A deeper understanding of host-gut microbiota interactions would require both advanced tools to analyze the diverse gut microbiota in a more dynamic manner as well as more advanced and physiologically relevant models [34]. Metagenomics, metametabolomics, metaproteomics, and metatranscriptomics that are enabled by high throughput and multiplex biosensing units are now powerful tools to investigate the composition of gut microbiota associated with host diseases [35]. In parallel, human intestinal organoids and gut-on-chip technologies represent two recent breakthroughs in modeling microbiota-associated human diseases *in vitro* [36, 37]. These technologies are in their early stages and would benefit from the incorporation of advanced materials that could assist in sampling, isolation, analysis, and biosensing of all components of the gut microbiota and would benefit from the greater incorporation of hydrogels and microfluidic devices that more closely represent human gut physiology.

Beyond their close association with human diseases, the gut microbiota also has beneficial effects on host health [38]. For example, the gut microbiota protects animals with type 1 diabetes (T1DB) [39]. Nonobese mice with irradiated gut microbiota are usually more susceptible to T1DB diseases. However, once germ-free mice are exposed to microbiota from healthy mice, the risk of T1DB development is significantly reduced. There have also been both studies and/or anecdotal reports on the beneficial effects of whole community microbial transplantation on treating human diseases including inflammatory bowel diseases (IBD; Crohn's disease or ulcerative colitis), chronic constipation, and Clostridium difficile-induced diarrhea [40, 41]. Taken together, research over the past few decades has revealed that gut microbiota is associated with differences in host health: dysbiosis of microorganisms in the gut may cause diseases (or may result from it), while implantation of healthy gut microbiota may restore intestinal homeostasis.

The aforementioned literature showing that the gut microbiota may modulate human disease has inspired effective approaches to manipulate gut microbiota composition, functions, and signaling pathways. Materials that can facilitate the efficient oral delivery of synthetic microbiota or drugs that regulate microbial pathways are thus critically needed, with a strong consideration of the biochemical barriers (e.g., strong acids and enzymes) that exist in the GI tract [42–45]. When microorganisms are delivered without any protective materials, their functions will be largely compromised [46, 47]. In addition, although it has not yet been demonstrated, it is highly likely that materials that enable microbiota to be delivered in region-specific, temporally controlled, and/or stimuli-responsive ways could also be desirable, given the high spatial variations and the dynamic nature of the gut microbiome. Dietary materials, including fibers, represent a specific group of materials that can remodel the gut microbiome [48]. Manipulating the gut microbiome through diet has already been a promising direction for treating many metabolic diseases, including obesity [49]. However, because of the high complexity of diets, it remains a major challenge to understand the relationship between the compositions and functions of dietary materials and the effects on gut microbiota. Answering these questions, however, will facilitate the better design of materials and therapeutics for treating gut microbiome-associated diseases.

The effects of the human microbiome on host health and diseases are mediated not only by direct actions of the microbiota but also through their effects on drug metabolism [50]. Although the human genome has been linked to drug responses with the emergence of personalized medicine, the link between drug response with genes associated with gut microbial symbionts has only become evident in the past decade [51]. There are many types of drugs known to be affected by gut microbes. For example, analgesics such as nonsteroidal anti-inflammatory drugs (e.g., nonsteroidal anti-inflammatory drugs or NSAIDs) were previously found to cause various levels of damage in the small intestines depending on the differential activities of microbial communities present at the time of drug administration [52]. Specifically, a higher population of gram-negative bacteria was found to be associated with greater damage. Probiotics have thus been studied for the prevention of NSAIDs-associated small intestine inflammation [53]. Similarly, antibiotics, cardiac glycosides, and metformin have all been reported to demonstrate an alteration of activity in response to the diverse gut microbiota-associated conversion of the drug molecules [54]. Bacterial CYP450 enzymes and other enzymes necessary for the reduction, hydrolysis, dihydroxylation, dealkylation, demethylation, decarboxylation, acetylation, deamination, and deconjugation of the gut microbiota are often responsible for the modification of drug molecules and, as a consequence, differential host responses [55, 56]. Elucidation of how the gut microbiota alters drug metabolism will thus further instruct the design of oral drug delivery systems by either the design of prodrug approaches or by avoiding the enzymatic conversion of drug molecules.

In summary, there are still major questions regarding the study, analysis, and manipulation of the gut microbiome, which would be greatly facilitated by synergizing these efforts with discoveries from advanced material engineering. The following sections provide an overview of the microbiota-gut-brain axis and then a summary of existent material-facilitated gut microbiome studies. Next, a discussion is provided on how gut microbiome research could also provide new strategies for material research. This multidisciplinary approach can contribute to better treatments for gut microbiome-associated chronic diseases through the collaboration of bioengineers, biologists, material scientists, chemists, and clinicians who treat these diseases.

### 2.2. Microbiota-Gut-Brain Axis

**Figure 2:**
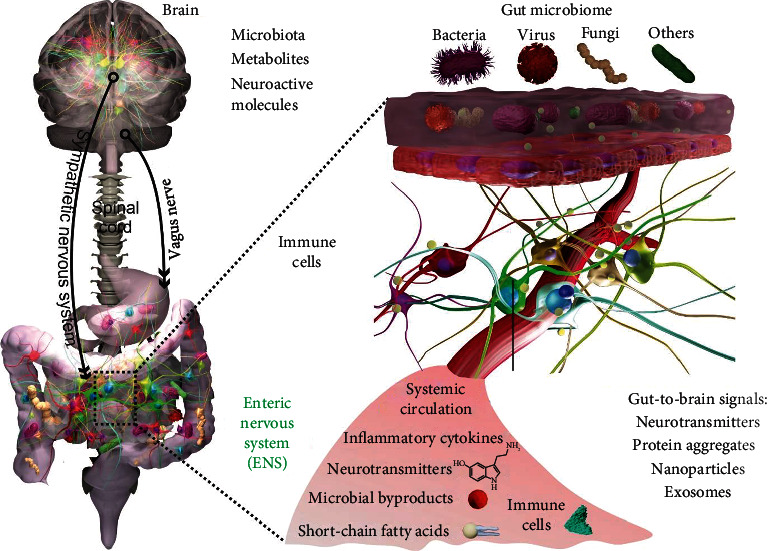
The microbiota-gut-brain (MGB) axis. Brain signaling can both regulate and be influenced by the gut microbiome, through intermediate gut mucosa, enteric nervous system, and immune system or by systemic circulation of gut microbiota-regulated factors. In parallel, multiple gut-to-brain signals, including neurotransmitters, protein aggregates (e.g., *α*-synuclein and *β*-amyloid), nanoparticles, and exosomes can also mediate gut-to-brain communication. Alteration of the gut microbiome can disrupt normal gut-to-brain signaling and has been associated with multiple neurological disease pathways, as shown at the bottom left.

The gut-brain axis is defined as the continuous, bidirectional communication that occurs between the GI tract and the brain (the central nervous system; CNS) (Figure 2). This communication can occur through multiple types of interactions, largely including the gut microbiota and/or neurotransmitters and other mediators that the microbiota often modulate. The microenvironment that the gut microbiota inhabit is in a symbiotic relationship with each component of the intestine, including its nervous system, known as the enteric nervous system (ENS), as well as its protective coat, the epithelium. Together with the CNS, the gut microbiota and these intestinal components are constantly in communication with one another. Alterations in the gut microbiota can thus affect both ENS and CNS plasticity and functions, including mood as well as GI motility and secretion [57–59]. Research has increasingly shown that these interactions, under normal conditions, serve to maintain both gut and brain homeostasis [60–62]. When any of these systems go awry, these interactions can result in brain and/or gut dysfunction and/or disease [58]. A major class of disorders that can result from these atypical interactions are disorders of gut-brain interactions (DGBIs), including irritable bowel syndrome (IBS). Mood disorders, like anxiety and/or depression, have also increasingly been shown to be affected by this axis [63, 64]. Critical microbiome modulators include diet, probiotics, and medications (e.g., antibiotics). Not surprisingly, these factors have been repeatedly associated, in both clinical and preclinical studies, with DGBIs and mood disorders [63, 64]. For example, different strains of probiotics, including Bifidobacteria and lactobacilli, have been associated with positive effects on anxiety and cognition in both animal models and humans [58]. Extending outward from the immediate gut environment is a key part of the autonomic nervous system that serves as “highways” to facilitate the continuous, bidirectional communication between the gut and the brain. Although the vagus nerves are studied the most, sympathetic signaling has also been shown to be potentially important [65–67].

Preclinical animal models and particularly animals that are raised germ-free (GF; completely devoid of microbiota) or those exposed to broad-spectrum antibiotics that eradicate the gut microbiota have elucidated important roles for gut microbiota in various facets of development, health, and disease [60–62]. Although GF animals appear healthy and fully functional, they develop significant anatomical and physiological abnormalities, particularly in the enteric and central nervous systems, when compared to conventionally raised animals [67]. These differences include alterations in ENS development, motility, and gut barrier function [60, 62, 68]. GF mice also display abnormalities in metabolic, circulatory, and endocrine function as well as mood and behaviors, including anxiety, stress, and social interactions [62, 63, 68]. There are, however, significant translational limitations to the study of GF animals, as no human is born lacking microorganisms and/or remains fully contained within a completely sterile environment throughout life. Moreover, abnormalities in GF mothers can impact fetal development [69]. Thus, interpreting microbial influences on development of these offspring may not be fully representative of normal fetal development [70].

Broad-spectrum antibiotics have been utilized to yield a “pseudo” GF environment in conventionally raised animals or to alter specific microbial communities, to provide mechanistic insights into microbial-host interactions and have thus afforded a complementary approach to the study of GF models [67, 71–73]. To further validate the role of the gut microbiota in gut and brain plasticity and functions, investigators have also utilized fecal microbiota transplant (FMT) as a mechanism to target the effects of specific microbe recolonization in either antibiotic-treated or GF animals [59, 60, 67, 71]. FMT reestablishes a microbial system from fecal bacteria obtained from a “healthy” donor into a diseased model and has been successfully utilized in cases of recurrent Clostridium difficile (C. diff) infection where current therapeutic interventions have been less successful. Clinical trials of FMT in humans are ongoing for individuals with neurodevelopmental, metabolic, and CNS-focused disorders like autism spectrum disorders (ASD), obesity, and Alzheimer's disease, respectively [74].

### 2.3. Byproducts of the Gut Microbiota That Influence the Gut-Brain Axis

There is increasing evidence to suggest that metabolites of the gut microbiota facilitate microbial-host interactions and, conversely, that the microbiota itself interacts with host epithelial and immune cells to release neurotransmitters or initiate signaling pathways involved in neuroimmune homeostasis (Figure 2) [75–77]. For example, microbial metabolites such as short-chain fatty acids (SCFAs), tryptamine, bile acids, dopamine, gamma-aminobutyric acid (GABA), and serotonin have been shown to influence local functions in the GI tract, including in the ENS, and also have far-reaching effects on the CNS [66, 75, 76, 78]. Transmitters, including GABA, dopamine, tryptamine, and bile acids, have been comprehensively described in other reviews [75, 76, 78]. We examine two well-studied modulators, SCFA and serotonin.

#### 2.3.1. Short-Chain Fatty Acids

Diet has a profound impact on the composition and activity of the gut microbiota. SCFAs, which are byproducts of bacterial fermentation of dietary nondigestible carbohydrates, including fibers and resistant starch, have been identified as important modulators of the MGB axis [79]. Oligosaccharides, a type of indigestible carbohydrate, has been shown to enhance SCFA levels with resultant beneficial effects on GI barrier function as well as postnatal ENS development [80]. Oligosaccharides also serve to influence signaling pathways within the CNS that regulate stress, with resulting improvements in anxiety and depression in both mouse models and humans [79]. In GF mice, treatment with acetate, propionate, and butyrate, the three major forms of SCFA, lessened the normally intensified stress-associated morphological, behavioral, and physiological functions previously observed in these mice, highlighting the importance of microbial-derived SCFA in regulating the gut-brain axis potentially via the major stress pathway in the body, the hypothalamic-pituitary axis [81].

In addition to prebiotics from diet, probiotic manipulation of the gut microbiota also produces SCFA [82]. The most well-studied probiotics include lactobacilli, Bifidobacteria, and bacteria from the Ruminococcaceae and Lachnospiraceae families, with lower levels of the latter implicated in patients with inflammatory bowel disease (IBD; ulcerative colitis and Crohn's disease) thus emphasizing a potential role for SCFA in the regulation of intestinal inflammation [83]. This has sparked interest in targeting SCFA biosynthesis as an IBD therapeutic by probiotic and/or prebiotic administration or formulating drugs that can elevate specific SCFAs, such as butyrate, that have been associated with IBD pathogenesis [84].

#### 2.3.2. Serotonin

Serotonin (5-HT) is a neurotransmitter essential for both CNS and ENS development and function. Abnormal serotonergic signaling is implicated in neurodevelopmental and mood disorders, including autism spectrum disorders (ASD), anxiety, and depression [85, 86]. The GI tract is where the body's largest depot of 5-HT is synthesized with the overwhelming amount synthesized in the intestinal mucosa. The homeostasis of intestinal mucosal 5-HT is regulated in part by the gut microbiota. For example, spore-forming bacteria can increase the production of 5-HT in the colon and serum of GF mice which can subsequently influence GI motility [71, 87]. This increase is likely mediated by the direct action of SCFAs on tryptophan hydroxylase (Tph1), the rate-limiting biosynthetic enzyme of mucosal serotonin, resulting in increased 5-HT biosynthesis [78]. Upstream of 5-HT biosynthesis, the gut microbiota can also influence the synthesis of the 5-HT precursor, tryptophan [88]. Microbial-mediated tryptophan catabolites, including tryptamine and indole, have been found to influence various host physiologic processes involving the immune system, GI barrier function, and GI motility [88]. In GF mice, 5-HT biosynthesis and metabolism are increased, implicating these processes as microbiota-driven [62]. Interestingly, GF mice also exhibit anxiolytic and anti-depressive-like phenotypes, and conversely, the depletion of tryptophan exacerbates a depressive-like phenotype, highlighting the potential importance of microbial-associated 5-HT and its signaling molecules, in the regulation of mood disorders [62].

Microbial-serotonergic signaling has been shown to be bidirectional. In addition to the ability of the microbiota to influence 5-HT signaling, gut mucosal 5-HT can also directly regulate colonization of microbiota, specifically *Turicibacter sanguinis* to consequently affect host physiological processes, including lipid metabolism [77]. The ability of *T. sanguinis* to recognize and respond to 5-HT is likely facilitated by a component within the bacteria that closely mimics the serotonin reuptake transporter (SERT), a transporter present in gut epithelial cells, and also neurons of the ENS and CNS, which is employed by host cells to take up 5-HT, leading to its intracellular inactivation [77].

## 3. Materials-Enabled New Models for Studying Human Gut Microbiome

Due to the limited access to human gut tissue, current gut microbiome studies have relied largely on *in vivo* animal models [89]. Although these models recapitulate several vital aspects of human physiological systems, *in vivo* animal models have restrictions for understanding the effects and mechanisms of dietary, drug, and other factors in the development of the human gut microbiome and during disease treatment [90, 91]. This is because human and other animal species have distinctive diets, and animals may respond to drugs differently due their distinctive enzyme pools [92]. In this regard, *in vitro* models of the human gut microbiome could offer several advantages [93]. First, human cells, including intestinal cells, colonic cells, and microbiota derived from human patients, can be integrated into *in vitro* models for a closer mimicry of human metabolism and host-microbiota interactions; second, as cells and culture processes are more standardized and can be better characterized *in vitro*, they are typically more reproducible, which is critical for multicenter collaboration and scientific rigor [94–96]. Furthermore, *in vitro* models allow for noninvasive and real-time sampling of microbiota and host cells compared with *in vivo* animal models [97]. Therefore, it has advantages for the study of dynamic host-gut microbiota interactions. Lastly, compared to animal studies, *in vitro* cell culture would allow for more facile large-scale screening assays [98]. Despite the clear advantages for investigating mechanisms and screening therapeutics, the study of *in vitro* gut microbiome models is still mostly based on animal cells or 2D cocultures that have not yet succeeded in closely mimicking human gut tissues. Creating advanced human gut microbiome models with better mimicry of the biophysical (e.g., extracellular matrix), soluble (e.g., metabolites and growth factors), and cellular (e.g., diverse microbiota) environment of human gut tissues would be critical for accelerating the discovery of novel microbial mechanisms and for the screening of signaling molecules essential in modulating host-microbiome interactions.

Material engineering has helped in important ways with the development of better gut microbiome models. This review points out critical gaps in research and highlights possibilities for how the different aspects of material engineering can facilitate advanced development of human gut microbiome models.

### 3.1. Stem Cell Reprogramming and 2D Epithelial Differentiation

2D enterocyte models, including monolayer intestinal cultures, have been widely used for drug metabolism and gut development studies since the early 1980s [99]. Their coculture with microbiota has also been considered a useful tool for studying the gut microbiome [100]. However, most of the enterocytes have been derived from mice and other animals, which do not recapitulate some of the key human physiological conditions. While primary human enterocytes can be harvested from patients or healthy subjects, invasive procedures are typically required, and a limited number of enterocytes can be obtained from each procedure. Stem cells, especially induced pluripotent stem cells (iPSCs) and intestinal stem cells, are characterized by their ability to proliferate and differentiate [101]. Therefore, stable lines of human enterocytes can be obtained from stem cell culture. The efficiency and speed of stem cell induction, expansion, enterocytic differentiation, and maturation, however, are crucial considerations for both clinical applications and fundamental studies [102]. Current protocols for the expansion and differentiation of stem cells have mostly focused on the optimization of soluble factors, such as media formulation. However, biophysical cues mediated by the extracellular matrix are known to regulate a variety of stem cell behaviors [103, 104]. Therefore, it is reasonable to expect that materials will play an important role in the maintenance, expansion, and differentiation of stem cells and the maturation of the differentiated enterocytes, which can eventually lead to more translatable and functional clinical models for investigating the gut microbiome [105]. Meanwhile, most current intestinal stem cell culture systems rely on static and simplified culture conditions that sometimes do not represent the crucial dynamic environment existent in the human gut. For instance, alongside mucin gels in the gut, there are multiple gradients, including ECM compositions, growth factor concentrations, cytokine types, oxygen, and pH [106] . Currently, very few materials have been able to provide a precisely controlled gradient microenvironment, representing a future direction for biomaterial innovation. For example, a recent study by the Allbritton group used a novel magnetic nanoparticle-integrated substrate to create a dynamic friction system to enhance the maturity of a differentiated colon epithelium layer, which is characterized by increased expression of MUC2, IL8, and E-cadherin in a transwell setup, compared with static culture [107]. Nevertheless, this direction is still at its early stage, leaving considerable room for material scientists and bioengineers to explore.

Biomaterials have demonstrated potential for accelerating the induction and expansion of stem cells as precursors of gut microbiome models [108]. iPSCs, embryonic stem cells (ESCs), and intestinal stem cells are three stem cell sources that are commonly used for deriving *in vitro* gut cell culture models to study the gut microbiome [109]. While ESCs and intestinal stem cells are typically harvested from human subjects, the generation of iPSCs requires reprogramming of somatic cells such as skin fibroblasts [110]. Although the process of generating iPSCs typically involves less ethical issues compared to ESCs and is less invasive than harvesting intestinal stem cells, rapid and efficient reprogramming of somatic cells into iPSCs is challenging, restricting the throughput in screening studies. In addition, recent reports have suggested that stem cells can lose their pluripotency during prolonged culture in the petri dish, which can result in inefficient differentiation into gut cells when they reach a high passage [111]. Advanced biomaterials that can support the expansion of stem cells without losing their pluripotency would thus be highly desired. Lastly, an in-depth understanding of the gut microbiome requires combinatorial studies, and the ability to generate sufficient gut tissues from stem cells would be beneficial.

Biomaterials can also regulate the *in vitro* reprogramming of somatic cells into iPSCs by providing more efficient gene delivery in a nonviral manner. Conventional approaches have relied on lentivirus- and retrovirus-based transduction of reprogramming genes (e.g., Yamanaka factors), which raises concerns of off-target effects including oncogene activation and often encounters challenges in large-scale production [112]. Nanoparticles, including chitosan nanocomplexes, polyethyleneimine-coated iron oxide nanoparticles, and liposomes, can provide nonviral gene delivery for the reprogramming of somatic cells into iPSCs [113]. From a previous study, 60% of the iPSCs derived from nanoparticle-based transfection are free of exogenous DNA integration in their genome [114]. Despite clear advantages, the efficiency of nanoparticle-based gene delivery and iPSC reprogramming is still not as high as viral-based approaches [115]. The efficiency of nanoparticle-based iPSC reprogramming can be further enhanced, however, by modulating cellular uptake mechanisms (e.g., clathrin- and caveolin-dependent endocytosis and micro- and macropinocytosis), endosomal escaping (e.g., through proton sponge effects or using thiolated or selenium-modified nanocarriers), and nuclear trafficking [116].

Additionally, biomaterials that amplify biochemical and/or biophysical signaling have also been applied to guide iPSC reprogramming and ESC pluripotency maintenance [117]. The effects of surface ligands, ligand densities, stiffness, and nanotopographies of ECM on stem cells and iPSC reprogramming are widely recognized [118]. For example, when substrates are modified with vitronectin-derived heparin-binding peptides, fibroblast adhesion to substrates is enhanced, leading to better proliferation and maintenance of the pluripotency of iPSCs during prolonged culture and passaging [119]. Biomaterial topographies also play an important role in iPSC reprogramming [120]. For instance, aligned nanofibers and microgrooved substrates were found to accelerate the conversion of fibroblasts into iPSCs (Nanog+ colonies) [121]. Topography-mediated alteration of cytoskeletal remodeling and epigenetic modifications, such as treatment with an HDAC2 antagonist or upregulation of WDR5, eliminated the effects of microgrooved substrates on iPSC reprogramming [122].

The differentiation of iPSCs, ESCs, and intestinal stem cells into epithelium cells can also be regulated by biomaterials. From early embryonic development, mechanical forces mediated by cell-ECM and cell-cell interactions regulate epithelial cells, including differentiation, migration, invagination, and junction formation [123]. In a 2D cell culture, soft and laminin-rich substrates are essential to the expression of epithelial differentiation markers [124]. Also, Wnt signaling, which is known to be affected by substrate stiffness and nanotopographies, is essential for long-term intestinal stem cell culture, suggesting a potential role of biomaterials in the maintenance of stem cell pluripotency [125]. Additionally, fibrous silk fibroin has also been used as a substrate to induce corneal epithelial differentiation from iPSCs. A cell sheet with tight cell-cell interactions was further formed from differentiated epithelial cells on the same substrate [126]. Epithelial cells differentiated from iPSCs, ESCs, and intestinal stem cells have been used for the formation of tight junctions in studies on gut epithelium transport [127]. However, the effects of different stem cell sources and epithelial differentiation substrates on the tight junction formation and gut-microbiota interactions require further investigation. Also, many of the aforementioned protocols have not generated all subtype epithelial cells (e.g., endocrine cells) commonly existent in the human gut. Studies that focus on material-regulated gut epithelium differentiation from iPSCs and ESCs remain to be performed. Furthermore, it is worth noting that in addition to gut epithelium, several other cell types also play important roles in gut-microbiome interactions, including the enteric nervous system and the immune system [128]. Nevertheless, while the effects and significance of biomaterials on the differentiation of iPSCs and ESCs are confirmed, little work has focused on this direction. High-throughput screening using biomaterial libraries conveying different mechanical, compositional, and biochemical properties could be key to discovering novel materials for regulating stem cell reprogramming and gut cell differentiation [129]. Although individual components have been studied previously in material-directed stem cell behaviors, the incorporation of the study of different material properties, such as stiffness and topography, would require screening systems of much larger complexity and the integration of advanced analytic tools, like machine learning, to interpret the large-scale screening results. Lastly, as the derivation of iPSCs is still considered a time-consuming step, direct conversion of somatic cells (e.g., fibroblasts) into gut epithelial cells would be desired as well, but the potential role(s) that biomaterials play in direct gut cell reprogramming remains to be explored. The rapid, efficient, noninvasive, and consistent derivation of gut cells from somatic cells would, however, significantly accelerate high-throughput drug screening and identification of signaling molecules in gut-microbiome studies [130]. Future studies should thus focus on materials such as hydrogels with dynamic properties and more diverse biomolecules that have shown great potential in regulating these processes.

### 3.2. Mucin Gel in Gut Microbiome Signaling

As a crucial component in the gut microenvironment, the gut microbiota is constantly interacting with surrounding mucin gels, which act as a continuous barrier to pathogens and separate the gut epithelium from the microbiota [131]. Mucins, including those secreted by specialized gut epithelial cells (e.g., goblet cells), are a group of large glycoproteins with heavy O-glycosylation that oligomerize (oligomerization only occurs within MUC2, MUC5AC, MUC5B, MUC6, and MUC19) with each other through disulfide bonds to form the viscoelastic mucus [132]. Within this complex and dynamic polymeric network, there are antimicrobial molecules (e.g., specific and nonspecific immunoglobins) secreted by Paneth cells which keep the inner layer of mucus sterile [133]. Within the GI tract, mucus has a wide range of thickness from 700 *μ*m (in the stomach) to 150-300 *μ*m (in the small intestine), with a typical thinner inner layer that anchors to gut epithelium cells through transmembrane glycoproteins and a thicker outer layer that undergoes rapid turnover and constant degradation by anaerobic bacteria [134]. Mucus is viscous (compared to water), hydrated, and highly porous, allowing for diffusion of macromolecule (e.g., proteins, sugars, and essential nutrients) while blocking diffusion of biological particulates (e.g., bacteria) above critical sizes (~500 nm) [135]. For viruses smaller than 500 nm, mucus is still a strong barrier due to its viscous nature and the presence of virus-binding antibodies [136]. Mucus is a highly dynamic system and is responsive to microbiota or pathogen invasion [136]. For instance, when bacteria manage to penetrate to the inner mucus layer and bind to the cell-bound mucin, the extracellular domain of mucin will be cleaved by the pathogen and is released to prevent their further invasion to the host (e.g., H. pylori) [137]. This process is accompanied by the stimulation of secretion of mucins from goblet cells and inflammatory signals from microfold cells (M cells) [138]. Meanwhile, if the bacteria do not bind to the cell surface mucin, they will not be able to penetrate the inner layer because of a steric hindrance [134]. Certain microbiota, such as H. pylori, have developed strategies to bypass the mucus barrier by secreting mucin-degrading enzymes (e.g., glycosidases and haemagglutinin proteases (e.g., hapA)), increasing local pH, or through mucolytic activities that use mucin glycoproteins as energy sources [139]. These activities can collectively reduce the viscosity of mucin gel and dilute the immunoglobins [139] within. Some microbiota (e.g., viruses (e.g., HIV-1, poliovirus, and reoviruses) and bacteria (e.g., S. typhimurium, V. cholerae, and Shigella flexneri)) can also evade mucus by directly routing their uptake through the M cells, located in Peyer's patches of the small intestine [134]. Apart from the microbiota, there are other environmental stimuli in the gut that can alter the release of mucin, such as inflammatory stimuli (e.g., pathogen-associated molecular patterns or PAMPs, including prostaglandins and lipopolysaccharide), bile salts, cholinergic stimuli, vasoactive intestinal peptides, nitric oxide, and neutrophil elastase [140].

As described above, mucin gel can be considered an excellent naturally existent biomaterial that conveys signals between host and gut microbiome and can be modulated in response to various environmental stimuli [141]. Additionally, gut microbiota interaction with mucin gels is an essential part of host-microbiota communications [142]. However, our current understanding of microbiota-mucin gel interactions is incomplete. This is partially due to a lack of *in vitro* systems that fully recapitulate the dynamics and complexity of human mucin gels. Nearly all epithelium cocultures used in the field have relied on the use of malignant epithelial cell lines that present altered mucin-related genes, lack goblet cell-like properties, and secrete mucins with a different glycosylation [143]. It would thus be desirable to develop biological and biochemical approaches to generate dynamic and complex mucin gel systems [144]. For instance, when goblet-like gastrointestinal epithelial cells can be differentiated from human stem cells using soluble factors or biomaterial interfaces, we can expect a more representative mucin gel for the modeling of host-gut microbiota interactions [145]. Developing advanced methods that can analyze the dynamics of mucin gel in anaerobic conditions and with representative microbiota represents another opportunity for material engineers. Additionally, much of the knowledge scientists have already discovered regarding microbiota invasion inside the mucin gel can instruct the design of oral drug delivery systems, which have been discussed extensively in prior reviews [19]. Lastly, partially due to the difficulties of generating robust, complex, and physiologically relevant mucin gel *in vitro*, there remains a lack of understanding of how mucin gel structures impact microbiota migration, retention, growth, and pathogenic activation [146]. To overcome these challenges, there is a clear need for interdisciplinary research and collaboration among material scientists, bioengineers, and biologists.

### 3.3. Organoid Models

Minigut organoids, which include intestinal organoids, have revolutionized the way the human GI tract can be studied [147]. Gut epithelium-associated pathways have been typically studied using adenocarcinoma cell lines of epithelium origin that often demonstrate altered genetic information compared to native human intestinal epithelium [148]. Although there has been no single model that can investigate human GI biology as a whole, organ-like culture derived from stem cells, or organoids, are considered an elegant model as it can recapitulate the cellular diversity in a 3D structure and can expand *in vitro* long term. For example, gut organoids can include multiple differentiated epithelial cell types such as enterocytes, Paneth, goblet, and enteroendocrine cells and can be incorporated into systems with nonepithelial cell types, such as immune cells [149]. Gut epithelium and the associated mucin gel are the primary sites for gut microbial communities as well as major barriers for pathogen invasion into the GI tract [150]. The distinctive host-microbiota interactions in the human GI tract are typically not well reproduced in animal systems or *in vitro* models composed of unicellular epithelial cell lines. *Ex vivo* gut tissue culture harvested from the human intestine or colon has shown unique patterns representative of human gut-microbiota interactions, but this approach is only limited to short-term studies, and invasive procedures are required for tissue harvesting [151]. Gut organoids not only form multicellular tissue patterns but also incorporate critical human- and patient-specific factors, including age, sex, genetic background, and ethnicity, which are factors that may well shape the microbiota-host interactions [152]. Therefore, the development of advanced gut organoid model systems is critical for a better understanding of gut microbiome biology and has attracted enormous interest. However, compared with the real human gut, current organoid systems still lack an accurate recapitulation of all key physiological behaviors, including mucin. Therefore, novel engineering strategies are essential for constructing more advanced gut organoid cultures to overcome the current challenges.

Materials are essential components for the development of organoid technologies. *In vivo* development of the human GI tract starts from the endoderm at the embryonic stage and is mediated by interactions among epithelial cells, stromal cells, and the extracellular matrix (ECM) secreted by these cells. ECM not only provides physical support for the GI epithelium but also constantly remodels and modulates its functions and phenotypes [153, 154]. ECM in the GI is typically in the form of a network (>300 types of molecules) assembled from proteins (e.g., collagens, laminins, nidogens, fibronectin, elastin, and syndecan) and polysaccharides (e.g., hyaluronic acid) or glycosaminoglycans (GAGs) and proteoglycans (e.g., perlecan) [153]. Two major entities in GI ECM are the basement membrane and intestinal matrix. ECM is a key regulator of intestinal stem cell proliferation, crypt formation, and epithelium turnover [153]. Because of these important functions in the GI tract, ECM molecules are required for gut organoid development. Natural (e.g., decellularized ECM matrix) and synthetic (e.g., hydrogels) 3D biomaterials with well-defined properties are thus extremely useful for organoid culture. For example, the Engelbreth-Holm-Swarm (EHS) matrix decellularized from mouse sarcoma, also named as Matrigel®, Geltrex®, or Cultrex BME®, has already been widely used in organoid cultures of all types [155]. The EHS matrix contains collagens, laminins, fibronectins, and many other ECM molecules, as well as key biological components for organoid survival [155]. While the EHS matrix is excellent to support stem cell culture and growth, it suffers from several limitations that restrict its broad clinical applicability in understanding gut-microbiome interactions and developmental studies. As it is harvested from mouse sarcoma, the EHS matrix typically has high batch-to-batch variations, contains nonhuman and carcinogenic components, cannot be easily tuned with the properties, and also binds to specific growth factors (e.g., TGF*β*) that eventually alter the organoids [156]. The EHS matrix also has significantly (nearly an order) lower stiffness compared with ECM in the GI tract. Therefore, in order to study these reliably, there is a strong need for an EHS matrix alternative that is tunable, reliable, uniform, and reproducible. This field is at its very early stage, but the addition of both natural and synthetic matrices has already significantly contributed to studies of gut organoids.

**Figure 3:**
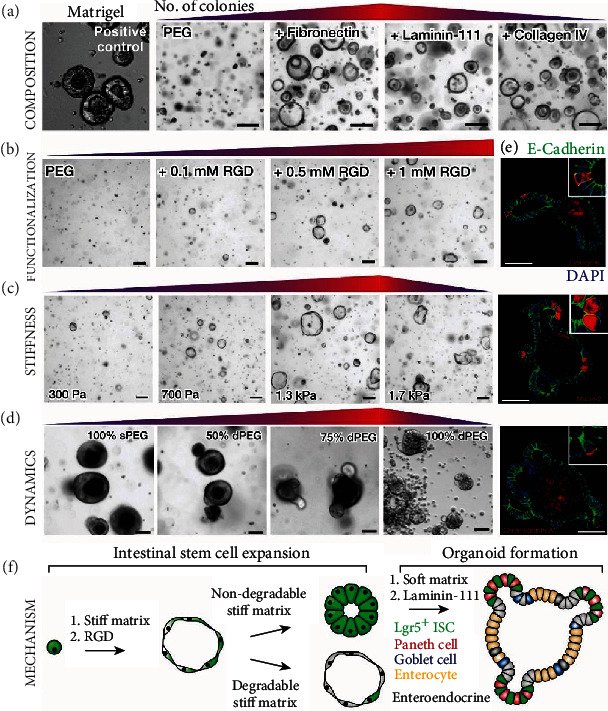
Synthetic materials for deriving intestinal organoids. (a–d) Effects of hydrogel composition (a), RGD functionalization (b), stiffness (c), and dynamics (d) on the proliferation of stem cells and formation of intestinal organoids. (e) Maturity of intestinal organoids formed from polyethylene glycol (PEG) hydrogel with optimal material properties. (f) Schematic explaining the mechanism of intestinal organoid formation within synthetic, dynamic hydrogels. Higher stiffness and RGD functionalization facilitate initial expansion of stem cells. At a later stage, however, a softer matrix better facilitates the differentiation and maturation of the organoids. Therefore, a dynamic hydrogel system that is stiff at the beginning followed by softening by degradation is desired for intestinal organoid maturation. Images adapted from reference [162], Nature Publishing Group.

Synthetic matrices for gut organoid culture have perhaps the best control of each material property such as composition, stiffness, viscosity, viscoelasticity, and adhesion ligand density [157] (Figure 3). Among the various types of synthetic matrices, polyethylene glycol (PEG) hydrogels have attracted the strongest interest thus far as they minimize nonspecific absorption of proteins and growth factors, thus excluding nonspecific interference. Additionally, precursors of PEG hydrogels can be tuned in terms of molecular weight, branches (e.g., single-arm vs. multiarms), crosslinking groups, and biodegradability, and most are commercially available. These precursors can also be further modified with signaling molecules and other biological ligands as a biochemical niche. Earlier research has shown that the mechanical properties of PEG hydrogels can be precisely tuned for the culture of intestinal organoids [158]. During the initial stages of organoid formation, stiffer (~1.3 kPa) PEG hydrogels are the most optimal for promoting the growth and expansion of organoids. At a later stage of organoid maturation, however, a lower stiffness (~190 Pa) in PEG hydrogel is desired. Mechanistic studies suggested YAP as a mediator for the differential responses of organoids to gel stiffness [159]. This study inspires the future design of mechanically dynamic PEG hydrogels to derive more robust intestinal organoids. Additionally, biochemical cues mediated by protein tethering to PEG hydrogels can also modulate stem cell behaviors in organoids. For instance, while PEG hydrogels functionalized with RGD do not support the organoid formation [160], additional functionality of laminin-111 leads to organoid formation [158]. Given the diverse signaling pathways involved in gut organoid development, dynamic hydrogels with switchable biochemical ligand tethering could offer significant advantages compared with EHS gels or conventional static synthetic matrices [161].

Hydrogels assembled from engineered peptides or recombinant proteins have also demonstrated different advantages for supporting gut organoid culture. Similar to PEG hydrogels, peptide or protein hydrogels are well-defined in terms of composition, offering better reliability over EHS gels for organoid culture [162, 163] (Figure 3). Additionally, engineered peptide and protein hydrogels can provide additional biological functions compared to PEG hydrogels. For instance, elastin-like proteins with both RGD and elastin domains have been engineered to form hydrogels that promote intestinal organoid formation. In these hydrogels, an orthogonal modulation of stiffness and RGD densities can be realized for the optimization of organoid culture. In a systematic screening, a study found that soft gel (stiffness: ~180 Pa) and higher RGD densities (3.2 mM) result in the most efficient expansion of intestinal organoids [164]. Immunostaining on organoids derived using elastin-like protein gel suggested a similar maturity compared to those derived from collagen I matrices. One crucial design principle for these protein matrices is the presence of MMPs in the culture [164]. The addition of MMP inhibitors prevented organoid formation. Therefore, understanding what levels of MMP levels are optimal for organoid culture would be important for the creation of more advanced protein matrices-based organoid culture systems.

Beyond the synthetic polymer, peptide, and protein matrices, matrices derived from natural biopolymers with more defined chemistry, including hyaluronic acid, alginate, and collagens, have also been used as a replacement for EHS gels. For example, murine intestinal organoids have been generated and maintained for over a year at an air-liquid interface, with high cell-type diversity including both fibroblasts and immune cells, in addition to epithelial cells [165]. Organoids cultured at the air-liquid interface showed gas transportation properties, which is critical for a large size 3D cell culture. The contractility of organoids can also be altered by the modulation of densities and geometries of the collagens inside the gel.

After deriving the gut organoids, host-gut microbiome interactions can be studied by colonizing the organoids with microbiota consortia. For example, human norovirus, which causes nearly 700 million cases of gastroenteritis annually, could not be cultivated in previous epithelial cell cultures or small animal models yet can be successfully grown in an intestinal organoid model with robust replications *in vitro* [162, 166]. Key characteristics of norovirus were also recapitulated in the organoid culture in a host-specific manner. For instance, organoids derived from individuals lacking genes encoding functional fucosyltransferase 2 enzymes showed reduced susceptibility to norovirus replication [166]. Such new understanding of host-specific pathogenesis obtained from gut organoid-microbiome cocultures thus have enormous potential for screening antiviral drugs. Other enteroviruses, including coxsackievirus B, enterovirus 71, poliovirus, and echovirus 11, have also been studied in regard to their replication, transcriptional regulation, and pathogenesis in human enteroids or organoids, and these studies have led to new findings above and beyond those shown using 2D culture of epithelial monolayers [167, 168]. For example, previous studies on viral coculture on 2D epithelial cell culture suggest that many of the aforementioned enteroviruses do not transfect gut epithelium but instead use them as mediators to transfect host cells in the internal organs [168]. However, 3D organoid models cultivated with this enterovirus suggest that epithelial cells can be directly transfected with enteroviruses, although the detailed mechanisms involved in this interaction remain to be explored [167]. Beyond virus-gut interactions, bacteria-gut interactions have also been studied using organoid systems. Intestinal organoids microinjected with salmonella strains showed upregulated expression of proinflammatory cytokines, including CXCL2, TNF, IL8, and IL1b, as well as factors that protect epithelium barriers [169]. Clostridium difficile, a bacterial species that causes infectious diarrhea, showed epithelium disruption in their coculture with intestinal organoids [170]. As a result, transporter-related reduction of mucus production and polarity were also observed. Although most of the gut microbiome studies in organoid culture have used EHS gels, one study used porous fibroin collagen hybrid gel for investigation of bacterial (e.g., Escherichia coli) invasion into an organoid and the associated immune responses [171]. This is an excellent example of how materials and organoids can facilitate pathogen-host studies.

Other than viral and bacterial interactions with GI organoids, the field is still at an early stage, as other species of gut microbiota, including archaea, protists, and fungi, have been rarely investigated. Also, in most cases, only a single viral or bacterial species that is already known for inducing pathogenesis was studied; however, the human GI microbiota is significantly more complex and dynamic. Furthermore, while synthetic matrices have shown enormous potential in the gut organoid culture, most current organoid-based studies on the gut microbiome have only used EHS gel, which poses limitations in reproducibility. Therefore, significantly more effort is required to develop and apply more advanced gut organoid systems for studies of the gut microbiome. In parallel, materials also have the potential to contribute to other aspects of organoid-based gut microbiome studies. For instance, 3D and 4D printed hydrogel-based multiwell systems have been applied for the generation of organoid arrays in the combinatorial screening of drug and other signaling molecules [172]. Given the high complexity and diverse signaling molecules in gut microbiome systems, we envision these multiwell systems being applied to high-throughput studies of the gut microbiome. Also, photoresponsive hydrogels have been used for the spatial patterning of stem cell-derived human tissues [172]. As GI tissues also show highly distinctive spatial patterns (e.g., duodenum, jejunum, and ileum in the small intestine), photoresponsive hydrogels could lead to the generation of more advanced, spatially patterned gut organoid models by providing different growth factors in different regions of the organoid culture. Interestingly, current stem cells used for deriving gut organoids have mostly been based on intestinal stem cells derived from small animals, or iPSCs, while human intestinal stem cells have been barely used in organoid studies.

Taken together, gut organoids provide an excellent platform for investigating human gut microbiome biology *in vitro*. However, the formation, growth, and maturation of gut organoids require a deeper understanding and better design of organoid matrices. Developing synthetic matrices or more chemically defined natural matrices as alternatives to EHS gels for organoid culture would allow scientists to investigate the biophysical cue-guided organoid formation and enable more accurate and advanced gut-microbiome studies.

### 3.4. Gut-on-Chip Models

3D gut-on-a-chip systems enabled by recent advancements in microfluidic fabrication have also emerged for modeling GI tissues [173]. Microfluidics not only provides *in vivo* mimicry fluidic flow but also allows for simulation of peristalsis, oxygen gradients, and pressure [174]. Additionally, compared to organoid systems, microbes can be more readily introduced for coculture of the gut epithelium in this 3D model [109]. The continuous and controllable fluidic flow not only more closely mimics physiological conditions of the human GI tract but also prevents the overgrowth of microbes that block the nutrient flow in regular insert-based cultures or 2D epithelial cultures [175]. Oxygen gradients are also critical for the maintenance of anaerobic microbial growth in the long term [176].

Materials can play a multifunctional role in enabling gut-on-a-chip systems. Earlier chips used for drug screening in the pharmaceutic industry have mostly adapted 2D epithelium systems cultured on 2D porous PET films, while the critical villi and crypt structures in human intestines are not well recapitulated [176]. Integration of villi-shaped scaffolding materials on the membrane addressed this challenge and created more biomimetic gut-on-a-chip systems [177]. Another parameter that differs from conventional gut-on-a-chip systems with the human GI tract is the stiffness of ECM in the culture [178]. Therefore, efforts have been made to integrate softer hydrogels as ECM materials for the culture of gut epithelium in the gut-on-a-chip systems [179]. For example, collagen hydrogels crosslinked by carbodiimide chemistry with a tunable stiffness of 20-2500 Pa have flowed into the microfluidic chamber [180]. Notably, upregulated expression of transporter proteins, as well as a higher transepithelial electrical resistance (TEER), was observed in this advanced villi-shaped, collagen gel-integrated gut-on-a-chip system, which is particularly interesting for pharmaceutical screening [181]. Efforts have also been made to incorporate peristaltic activities and oxygen gradients into the gut-on-a-chip systems [37]. Human intestinal tissues are characterized by lower oxygen levels than in the air, and oxygen gradients naturally exist in the mucus of the human GI tract [182]. Reconstructing such physiologically relevant oxygen gradients would provide a more sophisticated environment for the growth of microbiota. Earlier work on incorporating oxygen gradients into gut-on-chip systems was based on a multilayered microfluidic device that includes three serpentine channels separated by two layers of porous membranes with pore sizes of 1 *μ*m (for Caco-2 cell culture) and 50 nm (for supporting mucin and collagen gels), respectively [179]. Later on, the inclusion of bacteria microbiota into the microfluidics also successfully resulted in oxygen gradients [183]. Other directions for the development of gut-on-a-chip systems include the integration of biosensors (e.g., oxygen, pH, and glucose sensors) for real-time and *in situ* analysis of metabolic pathways [184].

**Figure 4:**
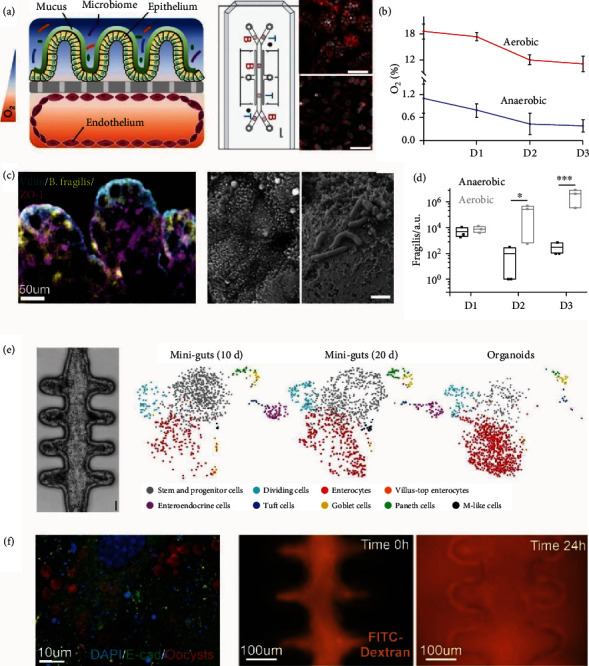
Gut-on-chip for modeling gut microbiome. (a) Design of the gut-on-chip device with microbiome, mucus, epithelium, oxygen gradients, and oxygen sensors. T: top layer; B: bottom layer. (b) Oxygen level measurement in the bottom channel of the chip showing the formation of an oxygen gradient in the mucus layer and anaerobic environment. (c, d) Growth of microbiota (B. fragilis) in the anaerobic environment of gut-on-chip (c) and comparison to nonaerobic environment (d). (e) Tubular minigut formed in a collagen gel embedded and laser bladed gut-on-chip device. Compared to normal intestinal organoids, tubular minigut has open lumen channels allowing for fluid flow and gut microbiota integration. Most importantly, miniguts showed higher maturity with enteroendocrine cells, M-like cells that are not normally existent in regular organoids. (f) Tubular minigut with gut microbiota (oocytes) seeded for the study of prolonged parasite infection (image on the left). Without infection (image in the middle), macromolecules such as FITC-dextran are not permeable through the epithelial layer. After long-term infection (image on the right), the gut epithelium becomes permeable, thereby recapitulating the *in vivo* disease pathology. Images adapted from reference [94].

More sophisticated gut-on-a-chip systems have also been applied for the investigation of the gut microbiome and host-microbiota interactions. Seminal work by the Ingber group demonstrated an intestinal chip that integrates oxygen gradients, oxygen sensors, and complex microbiota culture for advanced drug screening applications [94, 185] (Figures 4(a)–4(d)). Oxygen gradients were created by incorporating an anaerobic chamber and the addition of an endothelial layer into the device, and complex microbiota culture was derived from human stool followed by flowing into the chip [94]. Human ileum epithelial cells were also used in the model instead of conventional Caco-2 culture. In this way, they successfully extended the microbiota culture for over 5 days *in vitro*, which is a significant improvement compared with organoid-based microbiota culture systems (with continuous culture for about one day) [94].

Another innovative strategy toward more advanced gut-on-chip models is through the integration of organoid technologies [186] (Figures 4(e) and 4(f)). As mentioned in the previous section, gut organoids have the advantages of higher cellular complexity and diversity for the study of the gut microbiome. However, intestinal organoid culture typically results in a closed, cystic structure that limits the long-term culture, homeostasis, and coculture with complex gut microbiota [187]. Addressing this challenge, the Lutolf group used a tissue engineering approach to *in situ* form open 3D human intestinal organoid assemblies inside the microfluidic channel followed by culture with gut microbiota [188]. Because of the open-ended tubular structures, intestinal tissues can survive in the microfluidic culture for over a month. The colonization with microbiota induced anaerobic conditions that further matured intestinal tissues with rare cell types previously only shown *in vivo*. This concept of a functional gut-on-chip model enabled by extrinsic induction of stem cell self-organization may be able to be broadly applied to gut microbiome studies [189].

Microfluidics can also promote microbiota culture *in vitro*, even when not in the presence of gut epithelium [190]. High-throughput studies are typically required to optimize culture conditions for complex microbial communities to screen pH, oxygen levels, and media formulations [191]. Microfluidic arrays that are scalable, high throughput, and highly integrated are thus desirable for screening cultivation conditions for complex microbiota [192]. SlipChip, for example, allows for thousands of parallel single-cell cultures within connected microcompartments [193]. Cells were initially cultured in growth media to form microscale cultures, and then, different compartments in the SlipChip were separated into two parts, with one part applicable for analysis (e.g., taxonomic identification) and the other one for continuous culture [194]. Microfluidics has also been used for the identification of syntrophic partners in microbial growth and phylogenetically diverse microorganisms, using similar high-throughput screening approaches. Although these technologies have not yet been combined with gut-on-a-chip systems, they have revolutionized researchers' understanding of the diverse microbial activities of microorganisms [195].

Gut-on-a-chip systems have already led to discoveries that were not possible in previous *in vitro* models [18]. For instance, an anaerobic intestinal chip revealed that significantly enhanced growth of anaerobic microbe genus Akkermansia is closely associated with better preserved epithelial barriers [94]. However, it is well agreed that current models remain to be improved to better recapitulate the physiological conditions of human gut tissues. Continuous engineering of gut-on-a-chip in terms of better mimicries of pH, oxygen, metabolites, and combination with a full set of human microbiota would substantiate their potential for drug discoveries and numerous other applications. Additionally, there will be rapid development of more advanced multiorgan tissue models [196]. It would be critical to continuously improve knowledge of gut microbiome and human diseases by building sophisticated microfluidics emulating the human gut-liver, gut-immune, gut-heart, and gut-brain axis. For example, MINERVA, abbreviated from “MIcrobiota-Gut-BraiN EngineeRed platform to eVAluate intestinal microflora impact on brain functionality,” is a project recently funded by the European Research Council and is aimed at paving the road for the first multiorgan-on-chip system to emulate diseases associated with the microbiota-gut-brain axis. Although still at an early stage, its success will substantially transform human beings' knowledge of gut and brain-related diseases and lead to new approaches for drug development in treating neurological disorders. However, considerable amounts of work would be required to deliver the promise, which in turn offers excellent opportunities for biomaterials and biomedical engineering communities.

## 4. Materials for Analysis and Imaging of Gut Microbiome

The human gut microbiota is extremely complex, diverse, and dynamic [105]. Additionally, most species of human gut microbiota require highly specific culture conditions and have not been cultivated *in vitro* [197]. Therefore, a comprehensive analysis of gut microbiota and their communications with the host is thus extremely challenging. A variety of high-throughput technologies have thus been developed to address this challenge. For instance, metagenomics, metatranscriptomics, and metabolomics have provided excellent tools for profiling the gut microbiota [198]. Nevertheless, taxonomic composition alone is insufficient for understanding microbial pathways [198]. Critical information on the spatial and temporal distribution of microbiota species, genetics, proteins, metabolites, and their associated microbial pathways and host-microbiota interactions is still largely missing yet critical to the true understanding of human physiology [199].

### 4.1. Sampling Gut Microbiome

Material engineering has played crucial roles in various aspects involved in the analysis of the gut microbiome. The first step of gut microbiota analysis is typically the sampling and isolation of gut microbiota from human or animal samples [200]. The samples used for the sampling of gut microbiota have so far included feces, gut tissue biopsies, and the lumen of the gut [201]. Sampling methods have included direct isolation of microbiota from feces or using catheters, intelligent capsules, luminal brushes, or surgical tools to dissect gut tissues with gut microbiota [202]. All of the aforementioned methods have their advantages and limitations. For example, fecal samples are the most convenient for microbiota analysis, but they may not accurately reflect the composition and dynamics of microbiota in the gut as microbiota directly harvested from the gut using catheters, intelligent capsules, and/or luminal brushes [203]. The sampling efficiency of these latter methods, however, is limited. For example, ingestible microelectromechanical systems (MEMS) such as IntelliCap® from NIZO integrated with a fluid suction compartment, battery, and wireless communication unit allows for highly controllable sampling of GI fluids *in situ* [204]. However, the collected sample can easily leak out and become contaminated during travel within the GI tract, and microbiota in the inner layer of mucus may not be efficiently harvested [205]. Meanwhile, more conventional endoscopic aspiration methods, which are also the current clinical gold standard for monitoring abnormal bacteria growth in the GI tract, also share the same concern of contamination [206]. Developing smarter and better-controlled microoperation units may be a key to addressing this concern [207]. For instance, the future development of smart microrobots integrated with multichamber systems for spatially defined collection and the storage of GI fluids could provide excellent solutions [208]. Surgical methods that dissect gut biopsies can harvest microbiota samples and the associated gut tissues in their original structures and thus provide accurate and reliable profiling of microbiota species and study of microbial pathways at the sampling site [209]. However, because of invasiveness, biopsies are largely obtained from patients with underlying medical issues, thereby limiting their broad clinical applicability [210]. Equally invasive methods to obtain gut biopsies have included the luminal brush method that uses fiberoptic bronchoscope techniques, as well as laser capture microdissection techniques [211]. However, they still require advanced instruments and involve tedious procedures. Functionalized nanomaterials such as magnetic hydrogels, graphene, and iron oxide nanoparticles have also been recently developed for capturing microbiota in the GI tract [212, 213]. Particularly, because of their high surface area and small sizes, they potentially have high efficiency in penetrating mucus and binding to microbes both in the lumen and in the inner mucosal layer [214]. In particular, magnetic nanomaterials have distinctive advantages for the isolation of metabolites of microbiota through *in situ* binding followed by magnetic separation from the fecal samples, which have been combined with mass spectroscopy for microbiota metabolic profiling [215]. Nevertheless, because these technologies are still at an early stage of development, challenges still remain as to how to spatially control the capture of microbes and record the dynamics of diverse microbial species [216]. Most importantly, none of the methods can provide information on the intricate spatial distribution of the gut microbiome, which is critical for understanding spatially defined microbial pathways and interactions inside the GI tract.

**Figure 5:**
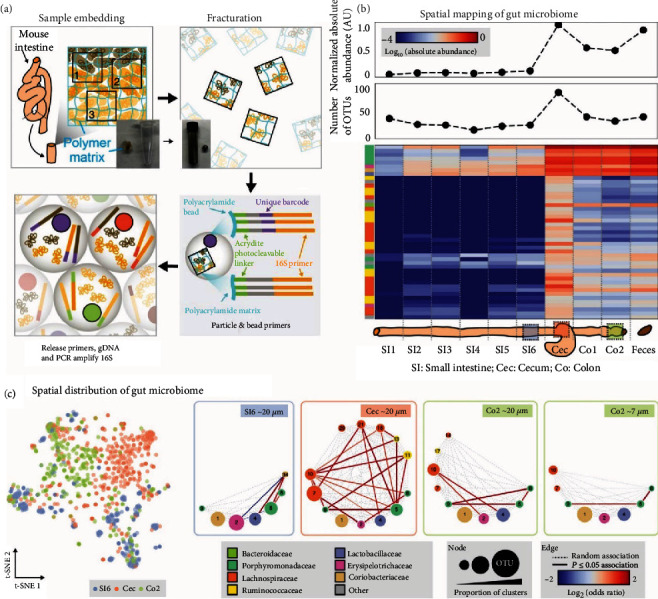
Sampling gut microbiome with preserved spatial information for spatial characterization of microbial biogeography. (a) Conventional methods for sampling gut microbiota in bulk often resulted in loss of spatial information that is critical for understanding the gut microbiome. In the MAP-seq approach, mouse intestine tissues were first dissected into different sections in a sequential manner; then, each section was embedded, barcoded, and fractioned for microbiota analysis through PCR and using 16S as a control gene. (b) Spatial map of intestinal microbiota. OTU: number of species. Heatmap is shown at the Log10 scale. Bray-Curtis dissimilarity was used for the clustering. (c) *t*-distributed stochastic neighbor embedding (*t*-SNE) visualization of Bray-Curtis OTU RA dissimilarity, showing the differential microbiota composition in different locations of the gut. The four graphs on the right are circular graphs of the pairwise spatial association for abundant OTUs. Nodes are correspondent to OTUs, and sizes are indicative of the prevalence of OTUs across clusters. Color indicates OTU taxonomy. Images adapted from reference [217], Nature Publish Group.

Sheth et al. developed a metagenomic plot sampling by a sequencing (MaPS-Seq) method to analyze the spatial organization of gut microbiota [217] (Figure 5). As a proof of concept, mouse small and large intestine samples were dissected and fixed by *in situ* crosslinking of a perfused acrylamide polymer precursor. The polymer precursor solution was also embedded with 16 rRNAs as an amplification primer commonly used in metagenomics studies [217]. After embedding, the tissue samples were then fractured without disrupting the spatial arrangement of the tissue. The fractured particles could then be captured by a microfluidic device followed by the release of genomic DNA for qRT-PCR analysis of taxa of microbiota information coupled with spatial barcodes [217]. This unbiased and accurate spatial metagenomics approach provides high-resolution (~20 *μ*m) analysis of the gut microbiome and provides definitive proof of the high spatial heterogeneity of gut microbiota. For instance, this study discovered that positively coassociated taxa, particularly for Ruminococcaceae, Lachnospiraceae, and Porphyromonadaceae, are present in the cecum, while the ileum is characterized by fewer taxa associations in the network. Although this work established an innovative framework for spatial metagenomics, the reconstruction of high-resolution spatial information within each part of the gut relies on advanced data analytic tools that can be further improved by the integration of advanced tagging strategies. Additionally, capturing the dynamic host-gut microbiome interactions in live animals or humans also requires the development of noninvasive and real-time monitoring tools for the gut microbiota [218]. Therefore, there are abundant opportunities to further develop the gel-embedding and plotting strategy for diverse spatial profiling applications.

### 4.2. Imaging of Gut Microbiome

Advanced optical imaging techniques have also enabled the spatial and temporal analysis of gut microbiota [219]. Conventional gene and protein analysis methods such as fluorescent *in situ* hybridization (FISH), expansion microscopy, and immunostaining require tissue fixation postprocessing and are thus not suitable for live animal analysis [220]. However, the recent development of nondestructive *in situ* near-infrared (NIR) fluorescent labeling techniques has enabled the real-time monitoring of gut microbiota that provides both spatial and temporal information on host-gut interactions [221]. For example, Wang et al. used a click-chemistry-based approach by first feeding mice with a diet containing alkyne-labeled sugars (e.g., propargylglycine), which are supposed to partially replace polysaccharides in the bacterial membrane through sugar metabolism [222]. Afterward, an NIR-II fluorescent dye-containing azide group was conjugated to the alkyne-labeled microbiota after gavage of the dye. NIR-II fluorescent dye is beneficial for gut imaging because of the high depth of gut tissues and the relatively low scattering of NIR-II laser by tissues. This study tracked the dynamics of gut microbiota over a period of 11 hours [222]. However, this approach only allows for the analysis of overall microbiota without providing information on different species of microbiota. Also, this metabolic labeling method may not be applicable for viral species in the gut microbiota. Another report by Hudak et al. shows the expanded capacity of metabolic labeling by using two different biorthogonal chemistry (azide-alkyne and cyclopropane-tetrazine conjugation) methods to visualize capsular polysaccharides, lipopolysaccharides, and peptidoglycans in three different color combinations [223]. However, the three-color combination approach only allows for *in vitro* imaging due to the strong scattering of red, blue, and green light by tissues [224]. Still, if only one specific biomolecule is needed for characterizing microbiota species, *in vivo* imaging and analysis of gut microbiota is feasible. Future directions to further develop this metabolic labeling strategy may include the integration of quantum dots, plasmonic nanomaterials, upconversion, and radioactive nanoparticles that allow for deep-tissue imaging (e.g., NIR fluorescence and surface-enhanced Raman spectroscopy) while having sharp emission profiles [224].

### 4.3. Analysis of Gut Microbiome

Microbiota analysis of samples harvested from feces, GI fluids, or gut biopsies has typically been based on metagenomics, metaproteomics, and more recently metatranscriptomics, which have already been comprehensively reviewed in the literature [225]. However, future development in new sequencing methods (e.g., nanopore) and microfluidic technology (e.g., single bacteria encapsulation systems) could advance metagenomics and single-cell analysis of the gut microbiome [226]. Current metagenomics has mainly used Illumina-based second-generation next-generation sequencing (NGS), which is not only time-consuming (sample to readout time around 2-3 days) but also limited to short-read sequencing [227]. Third-generation NGS, based on nanopore technologies, has enabled extremely fast (<6 hours) and long-read sequencing and has thus gained popularity in metagenomics analysis [228]. Given the recent advancement in graphene nanopore technologies, we envisage the continuous prosperity of nanopore-enabled genomic studies of the gut microbiome [229]. Similarly, in the past decade, there has been rapid development of advanced single-cell analysis technologies, from single-cell genomic, transcriptomic, and proteomic, to more recent colorimetric and epigenomic studies, with reduced costs and higher accuracy in single-cell sampling, encapsulation, and barcoding through advanced microfluidics design and software integration [230]. Although their applications in microbiota analysis are expected to significantly facilitate the discoveries of dynamic and heterogenous microbial pathways that cannot be achieved by bulk genomic studies, the translation of single-cell techniques into gut microbiome analysis has been limited [231]. Critical barriers include the extremely large population, as well as the smaller sizes, of gut microbes. The large number of cells will require improvement of microfluidic devices in terms of reliability during continuous sample processing and also adds significant challenges in terms of data analysis. To address this challenge, the recent development of the CRISPR-cas13 biosensing platform provides a promising tool for multiplex detection of RNAs [232], as compared to the previous qRT-PCR, loop-mediated isothermal amplification (LAMP) approaches, but how to incorporate CRISPR-cas13 into droplet systems for digitalized biosensing remains elusive [233]. More importantly, the sizes of water-in-oil droplets used for single-cell encapsulation in microfluidic devices may need to be decreased in cell size to reflect eukaryotic cells to bacteria [234]. Reducing the channel size alone may lead to higher chances of failed encapsulation and more miniaturized droplet sensing units [235]. Therefore, there will be challenges but also many opportunities to develop advanced microfluidic devices for single-cell technologies in gut microbiome analysis.

### 4.4. Biosensors for Gut Microbiome-Associated Diseases

**Figure 6:**
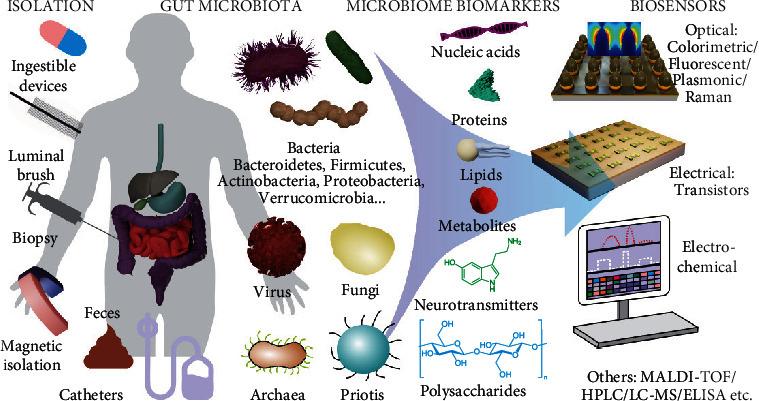
Materials for isolation and analysis of gut microbiome. Different methods have been developed for the sampling of gut microbiota, which include bacteria, virus, fungus, archaea, and priotis. Biomolecules associated with gut microbiota, including nucleic acids, proteins, lipids, metabolites, neurotransmitters, and polysaccharides, can be used as biomarkers for the quantification of microbiota species. Biosensors that can be used for gut microbiota detection include optical, electrical, electrochemical, and other approaches.

Developing sensitive, selective, multiplex, and point-of-care (POC) biosensing units can also significantly facilitate the analysis of the gut microbiome and their influence on host health and disease status (Figure 6). Although metagenomics is the gold standard for microbiome analysis, point-of-care biosensors that analyze crucial metabolites or disease biomarkers in fecal samples can accelerate the translation of findings in the gut microbiome into clinical diagnostics [236]. Compared to blood, fecal samples are easier to obtain and better suited for point-of-care diagnostics. Fecal biomarkers have been linked to a variety of diseases, including arthritis, cirrhosis, cancer, IBD, and metabolic and neurological diseases. For example, higher levels of branched-chain amino acids in fecal samples have been associated with insulin resistance in diabetic patients [237]. Many of the new biomarkers can be predicted by metagenomics or metaproteomics, which suggests an enormous opportunity for the future development of biosensors. For instance, a metagenomic comparison of subjects with Crohn's disease to healthy subjects suggested a higher population of facultative anaerobes in IBD-associated conditions [238]. This is further linked to higher levels of acylcarnitine, short-chain fatty acids, and bile acids and provides targets for the development of early-stage IBD biosensors [238]. In the past few decades, the material community has made strenuous efforts to develop various types of biosensors (e.g., electrochemical, plasmonic, and immunolateral flow assays and cantilever-based biosensors) previously developed for POC detection of proteins, small molecules, lipids, nucleic acids, and other biomarkers [239]. Given the identification of new biomarkers revealed by recent metagenomic and metaproteomic studies, material scientists have enormous opportunities to contribute to a variety of POC biosensors for analyzing host-gut microbiome interactions and monitoring host health status in human diseases.

## 5. Materials for Manipulation of Gut Microbiota

In the past decade, the gut microbiome has been widely associated with a variety of common and debilitating human diseases, including Parkinson's disease, Alzheimer's disease, diabetes, atherosclerosis, arthritis, and cancer [240]. As such, there has been immense interest in the medical, materials, chemistry, bioengineering, and biology communities to develop effective approaches for engineering and manipulating gut microbiota. In general, current approaches can be divided into suppressive or additive treatment based on mechanism-of-action (MOA). Suppressive therapies, typically mediated by antibiotics and based on specific or nonspecific inhibition of notorious bacteria, fungi, and viruses in the gut, are the most broadly adapted clinical strategies. Additive therapies, in comparison, are either based on the oral delivery of beneficial microbiota species (e.g., probiotics and genetically modified bacteria) or molecules (e.g., prebiotics) that augment the growth or metabolic activities of beneficial gut microbiota species [241]. Although direct oral administration of some of these additive or suppressive modalities has already led to effective therapeutics, challenges remain in how to improve their efficacy, achieve target specificity, and better understand the molecular mechanisms. Smart materials that can enhance the oral delivery of both suppressive and additive microbiome-targeted therapeutics may thus provide excellent solutions as the mechanistic underpinnings of bacterial functions, and interactions become more well understood. This section mainly discusses the new generation of microbiota modulatory approaches and highlights the opportunities for material engineering.

### 5.1. Suppressive Therapies

Smart materials have been applied for the delivery of various types of antibiotics, such as small molecules, peptides, proteins, and replicating entities to suppress microbes in the gut. Conventional antibiotics often target a broad spectrum of bacteria species even when only specific pathogens are causing diseases. This leads to the elimination of beneficial species in the gut microbiota, which has been associated with compromised outcomes in immunotherapy-based cancer treatment as well as other side effects. Furthermore, microbes treated with the same antibiotics repeatedly often develop resistance, ultimately leading to treatment failure. As such, an active direction of research is to develop more targeted antimicrobial approaches.

Engineered viruses, as parasites for selective killing of target bacteria, have been recently used for suppressive gut microbiota modulation, which is also known as bacteriophage therapy [242]. In nature, bacteriophages can infect the target and then replicate inside the bacteria, eventually leading to the death of the bacterial host. Unlike conventional small molecule-based antibiotics, bacteriophages can be engineered to incorporate capsid protein domains for enhanced binding to mucus or bacteria-derived biofilms [243, 244]. Thus, this engineered bacteriophage can have both better retention in the gut for prolonged antimicrobial applications and more specific targeting capability to certain microbiota communities [244]. In parallel, host adsorption factors can be engineered to change the target range of bacteria [245]. The killing of target bacteria, especially those that have developed resistance to conventional antibiotics or are protected by biofilms, can also be enhanced through genome engineering (e.g., CRISPR-Cas technologies) [246]. One critical barrier of bacteriophage therapy, however, is its limited efficiency in oral delivery due to the presence of a wide range of pH and proteases in the stomach and gut. Materials have been previously applied for the delivery of viruses, both through oral and parenteral routes in order to overcome these challenges; for example, a virus has been loaded into Escherichia coli for protected oral delivery into the host in the development of oral vaccines [247]. Viral-mimicking nanoparticles that enable oral delivery of plasmids have also been explored in diabetic treatments. However, whether these material-based approaches can be used to replicate the outcomes from bacteriophage-mediated therapies remains to be tested and thus represent a future direction for suppressive therapy in gut microbiome research.

### 5.2. Biomaterials with Therapeutic Functions for Additive Therapies

Biomaterials have been widely applied for the oral delivery of small molecules, peptides, proteins, and genes, with some of them having direct effects on the gut microbiome. Specifically, compared to parenteral drug delivery, oral administration is often safer and more convenient and has a faster FDA approval rate. However, because of the biochemical barrier in the stomach (e.g., low pH and various proteases), the mucosal and epithelium barrier in the small intestine, as well as the rapid clearance in the GI, oral drug delivery has typically shown lower efficacy compared to parenteral routes. Biomaterials, such as nanoparticles, microneedle patches, polymeric gels, and other ingestible devices, can address these barriers in oral drug delivery by encapsulating drugs, protecting them from aggregation or enzymatic degradation, enhancing GI retention, facilitating their penetration across the mucosal layers, and enabling tissue-specific drug delivery once entering systemic circulation [204, 248, 249].

Drugs that can impact the gut microbiome have also been delivered by biomaterials [45]. For example, antibiotics-loaded nanoparticles have been delivered orally with a more efficient sustainable modulation of the gut microbiome. Probiotics have also been delivered by microgels to improve host health and immunity. Beyond these studies, certain drug delivery biomaterials composed of natural biopolymers, such as glycans, proteins, and polysaccharides, may have direct impacts on the gut microbiome by acting as nutrients, suppressants, and boosters regardless of the type of drugs encapsulated [250]. For example, ginsenoside Rg3 functionalized iron oxide nanoparticles increased Bacteroidetes and Verrucomicrobia and decreased Firmicutes in gut microbiome, which was then found to associate with enhanced efficacy of treatment of hepatic cell carcinoma (HCC) [251]. One possible mechanism is that decreases in urea and 3-indolepropionic acid, as well as increased free fatty acid levels associated with alteration of gut microbiota, will lead to suppressed tumor growth. Another study used dextran-based nanoparticles to deliver irinotecan, an anticancer drug, while increasing probiotic bacterium C. butyricum to synergistically suppress colorectal tumor growth [252]. In addition, this nanosystem further combines phage for the selective inhibition and killing of F. nucleatum that are reported as a key inducer for chemoresistance in colorectal tumor. The same nanosystem also allows simultaneous incorporation of probiotic C. butyricum that further enhanced the antitumor efficacy. Given the high complexity of the gut microbiome and the complicated effects on host health, it has been a challenge to fully elucidate the mechanisms behind changes and predict the biomaterial-mediated modulation of the gut microbiome. The Kim group used a high-throughput screening approach and surveyed various safe food ingredients to investigate effects on gut microbiome [253]. From their screening, they successfully identified that gel formulated from inulin, a food polysaccharide component, and several bacteria including Akkermansia, Roseburia, and Lactobacillus were significantly increased and are associated with enhanced T cell immunity, especially for IFN-*γ*+CD8+ T cells during cancer immunotherapy.

In addition to enhancing immunity to cancer immunotherapy, a recent study from the same group also investigated nanoparticle-based modulation of the gut microbiome and IBD [254]. IBD is characterized by impaired epithelium barriers and inflammation which has also been associated with an altered gut microbiome. For instance, Clostridium XIV *α*, Akkermansia muciniphila, Clostridium coccoides, Lactobacillus, and Clostridium leptum are significantly decreased in a host with IBD. Actinobacteria, Proteobacteria, and Enterobacteriaceae on the other hand are increased which is directly related to the elevated intestinal inflammation. Although biomaterial-based anti-inflammatory drug delivery has been a widely exploited approach to mitigate IBD-associated inflammation, it remains unclear whether the gut microbiome is associated with the anti-inflammatory actions. Interestingly, a recent study reported a hyaluronic acid-bilirubin self-assembled nanoparticle that even without delivery of any anti-inflammatory drugs can mitigate the progression of IBD and intestinal inflammation. This suggests that biomaterials can often play a multifunctional role in the regulation of the gut microbiome. Given the large number of biomaterials that have been applied for oral drug delivery, it would be essential for future studies to understand the beneficial roles of biomaterials in the modulation of the gut microbiome, rather than simply treating them as drug delivery vehicles.

In addition to polymeric nanomaterials, inorganic nanoparticles have also been studied for their probiotic effects, even though their therapeutic functions through microbiome modulation are less explored. For instance, ceria nanoparticles have been previously studied as a nanozyme to scavenge reactive oxygen species which may promote the growth of bacteria. Also, ceria nanoparticles have also shown effects on the stimulation of the growth of Lactobacillus and Bifidobacterium species that demonstrate beneficial effects on obesity reduction and dyslipidemia. Interestingly, in vivo oral treatment of Balb/c mice with nanoceria leads to a significant reduction in blood cholesterol levels, but the detailed mechanisms remain unclear [255]. Selenium nanoparticles have also shown modulatory effects on microbiome that further leads to enhanced immune responses of host mice toward breast cancer cells. In parallel, while one report suggests that selenium nanoparticles stimulate the growth of Lactobacillus brevis, another report found the antimicrobial effects of selenium nanoparticles on Candida albicans. Given the vast array of inorganic nanoparticles developed in the past two decades, systematically understanding their therapeutic effects through modulation of the gut microbiome represents another challenge for the next decade's research.

### 5.3. Oral Delivery of Bacteria as Next-Generation Additive Therapy

**Figure 7:**
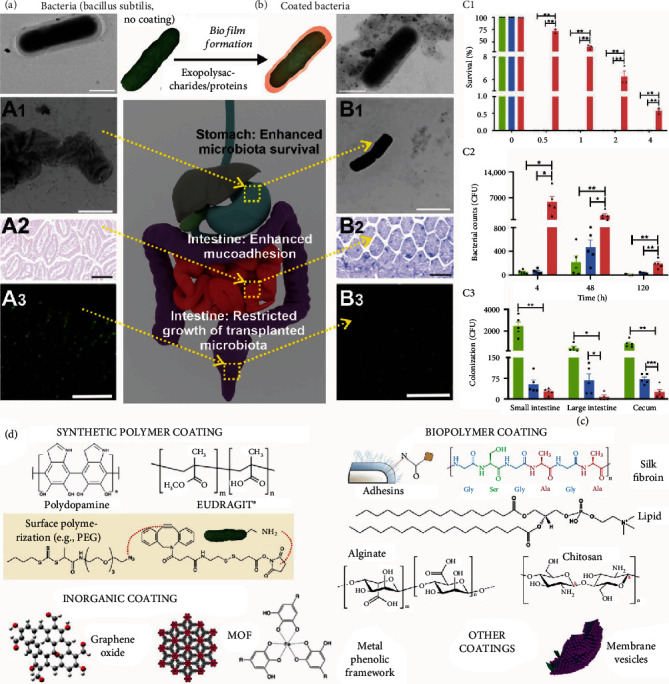
Oral delivery of coated bacteria for additive therapy. (a) Morphological comparison of naked bacteria (a, scale bar: 1 *μ*m) and biofilm-coated bacteria (b, scale bar: 1 *μ*m), and their acid stability (A_1_ and B_1_, TEM, scale bar: 1 *μ*m, quantification shown in C_1_), mucoadhesive properties (A_2_ and B_2_, gram staining, scale bar: 100 *μ*m, quantification shown in C_2_), and restriction of bacteria growth (A_3_ and B_3_, green indicates S. aureus staining, quantification shown in C_3_). (d) Three common strategies of bacterial coating for potential oral delivery applications and gut microbiota modulation, including synthetic polymers, biopolymers, and inorganic coating methods. Images adapted from reference [257], AAAS.

The discovery of the beneficial roles of human gut bacteria in maintaining human health has inspired many probiotic therapies that are mediated through oral administration of bacterial species such as E. coli, Bifidobacterium spp., and Lactobacillus spp. In parallel, bacteria can be engineered to express therapeutic proteins and other biomolecules. Additional efforts have also been made to evaluate engineered bacteria for *in situ* applications and prolonged release of recombinant proteins and other biologics to enhance their oral bioavailability and enable environmental stimuli-responsive on-demand delivery [256]. Therefore, harnessing the beneficial effects of bacteria with both natural therapeutic effects and the capabilities of releasing biologic drugs represent new paradigms in oral cell therapy and drug delivery. Through combined efforts from material scientists, bioengineers, biologists, and clinicians, the efficacy of orally delivered bacteria for the treatment of inflammatory, infectious, and metabolic diseases has been studied. Nevertheless, to achieve the ultimate goal of fully autonomous directed bacterial therapies, several challenges remain. First, personalizing bacteria species for the treatment of specific diseases requires significant advancement in metagenomics and metatranscriptomics as well as synthetic biology. Second, similar to oral virus delivery, achieving high bioavailability and retention of orally ingested bacteria in the low-pH and protease-rich environment of the GI tract remains a critical barrier that needs to be overcome [47]. Lastly, combining spatially controlled delivery of bacteria combined with temporally controlled release of biotherapeutics can enhance bacteria therapy, but how to implement this goal remains largely elusive given the complex and dynamic GI environment *in vivo*. Materials in the forms of pills, enteric coatings (e.g., Eudragit®), membrane polymerization, and gels have already been applied to address some of these limitations (Figure 7). Gene engineering approaches that increase the stress tolerance of bacteria and also enhance the survival of bacteria delivered orally are currently being studied [47]. However, their overall bioavailability remains to be improved, which can be achieved by more robust protection from the GI environment and *in situ* release in a spatially controlled manner and also by using smart biomaterials to enhance their retention in the GI tract.

Coating bacteria has become a promising strategy to address the challenges in oral bacterial therapy and microbiome engineering, as it provides protection in the GI tract against low pH and proteases, allows for rapid dissolution at the target location, and improves retention of bacteria in the GI tract through surface modifications [47, 258]. Some earlier work on coating bacteria was focused on energy applications by using bacteria as a source of photocatalytic reagents. Various types of semiconductor materials, such as CdS, TiO_2_, metal organic frameworks (MOFs), and silica, are successfully grown on bacteria membranes through surface-mediated nanocrystallization [259–262]. Among these coatings, biocompatible MOF coatings, such as Zr_6_O_4_(OH)_4_(BTB)_2_(OH)_6_(H_2_O)_6_; BTB = 1,3,5-benzene-tribenzoate, have been applied for biological applications [259]. However, these carboxylic coordinated MOFs may not survive in the low pH conditions of the stomach, which limits their applications in oral bacterial therapy. Polymer coatings are thus more attractive, but the surface chemistry to initiate polymerization is usually more restricted. For example, PEG hydrogels that are generally biocompatible but simple polymerize PEG precursors in the presence of bacteria may not result in an efficient coating. Therefore, photoinduced electron transfer- (PET-) based reversible addition-fragmentation chain-transfer (RAFT) polymerization was applied for the surface polymerization of bacteria [263]. 2-(Butylthiocarbonothioyl) propionic acid (BTPA) was selected for the metabolic modification of the yeast membrane, and biocompatible photoinitiator eosin Y was used to catalyze the reaction. Strikingly, this polymer coating still allows for the proliferation of yeast cells, with an exponential stage reached at 10 h. In addition, the surface coating is reversible and can be cleaved by strong reducing agents such as tris(2-carboxyethyl) phosphine (TCEP). Future applications of this coating strategy could lead to the development of versatile oral bacteria therapies, but the viability of bacteria with these coatings, under the hostile GI environment, remains untested. However, other formulations of bacteria with various types of coating have been tested for oral bacteria therapies. For example, calcium-crosslinked dioleoylphosphatidic acid (DOPA) lipids have been used for the protected oral delivery of Staphylococcus aureus (S. aureus) and Enterococcus faecalis (E. faecalis) with three times higher viability and enhanced retention in the GI tract (four times better than noncoated bacteria), respectively [264]. By further harvesting the therapeutic potential of orally delivered bacteria, the study demonstrated enhanced treatment of dextran sulfate sodium (DSS) and Salmonella typhimurium- (STm-) induced colitis through microbiome modulation. One potential issue for the liposome coating, however, is the instability of the coating in the GI tract when administered by oral gavage. The same group next developed a biofilm coating based on the natural phenomenon that bacteria can secrete proteins and exopolysaccharides such as TasA and BslA to form a tightly bound biocompatible film [257] (Figure 7). Using this biofilm coating strategy, Bacillus subtilis was orally delivered as a proof of concept, showing a 125-fold enhancement of bioavailability in a porcine model. Additionally, this biofilm can strongly interact with mucin gel in the gut, leading to a 17 times higher intestinal colonization rate and eventually modulating the gut microbiome effectively with a significantly lower population of Staphylococcus aureus. Nevertheless, current approaches are still limited in spatial control, which is critical considering that the microbiome has high variance in terms of microbiota.

Many efforts have been made to achieve region-specific oral bacteria delivery. For example, an enteric coating that protects bacteria at the low pH in the stomach but selectively dissolves in the higher intestinal pH (e.g., 5.5) has been applied for oral bacteria therapy [265]. Different locations of the gut, including both the small and large intestines, are characterized by their unique pH in the lumen. For example, the small intestine can be divided into duodenum, jejunum, and ileum, with pH around 6, 7-9, and 7.4, respectively [266]. Therefore, coating bacteria with pH-responsive polymers, such as Eudragit, can enable the spatially controlled exposure and anchoring of bacteria. Using this strategy, Feng et al. modulated the gut microbiome and enhanced the treatment of Salmonella-infected mice [267]. Another strategy to achieve spatial control of bacteria delivery has been based on the hybridization of bacteria with magnetic nanomaterials. By controlling the magnetic field, bacteria can then be localized to specific regions of the gut. One such approach is known as “cellular localization assisted by magnetic particles (CLAMP),” which has been used to deliver magnetic nanoparticle-labeled E. coli [268]. Although their spatial resolution is suboptimal in small animal models, only allowing accumulation of bacteria in the small intestine, they may be useful for human clinical trials given the larger sizes of the human GI tract and may also be suitable for targeting disease states limited to the small intestine. However, it remains elusive how to translate these technologies without requiring a special magnetic field generator.

In general, material-mediated oral bacterial therapy has shown excellent potential for treating gut microbiome-associated diseases by enhancing survival, bioavailability, and gut retention and facilitating on-demand and spatially controlled release. Given the species diversity in the gut microbiome, the enhanced oral delivery of multiple bacteria species using a coating or other scaffolding materials remains to be realized. Also, most current work has focused on the delivery and modulation of bacteria species in the gut. However, recent advancements in the study of the virome revealed the increasingly important role of viral species in gut microbiome-associated diseases. Using biomaterials for enhanced oral delivery of beneficial viral species may provide a new perspective on the role of viruses in homeostasis maintenance in the gut. Also, there has been a large number of studies that have applied materials for drug, protein, and gene delivery; both drugs and the delivery vehicles may have additive or suppressive effects on the gut microbiome, but most studies thus far have only focused on their therapeutic outcomes on host health. Incorporating gut microbiome analysis in future oral drug delivery studies would not only help identify the still concealed MOA of drugs but also lead to new therapeutic modalities for modulation of the gut microbiome.

## 6. Future Perspectives

### 6.1. Drug Delivery Approaches for Modulating MGB Axis

Several animal models and/or systems (e.g., antibiotic, prebiotic, or probiotic exposure, FMT, and GF) have been utilized to evaluate the role of the gut microbiota on gut-brain axis disorders and have lent evidence to the idea that targeting the gut microbiota may be a novel therapeutic target for treating gut-brain axis disorders, including DGBIs and mood dysfunction [63, 269, 270]. Utilization of these different models in concert is critical to understanding the true functions of the microbiota on these, and other, neurologic and/or psychiatric disorders. Before one can target specific communities, or even individual species, of gut microbiota, further research needs to be focused on the specific functions of individual bacteria and the impact of their interactions with other bacteria as well as with host factors. As these aspects are elucidated, a more targeted approach involving probiotics, prebiotics, genetically engineered gut microbiota, and dietary supplements can be implemented.

While studies evaluating FMT have yielded beneficial results in ulcerative colitis and C. diff infection, studies in other conditions such as Crohn's disease are largely preliminary and warrant further investigation. Other conditions where FMT is currently being examined include ASD and Parkinson's and Alzheimer's disease [63, 269, 270]. Future research areas that need to be addressed include examination of the effects of chronic microbiota-based therapies, region-specific targeting in the gut which is critical given that pH and oxygen levels differ across the length of the GI tract, and how the gut microbiota and generated modulators (e.g., SCFA and neurotransmitters) interact with the host. Further, the gut is also inhabited by a virome and fungome, both of which are likely to interact with the microbiome and require extensive investigation. Together, these factors are critical in the development of microbiota-based therapeutics targeting the MGB axis and will not only help the treatment of gut-brain axis disorders but also provide insights into microbial-host interactions that likely underlie their pathogenesis.

**Figure 8:**
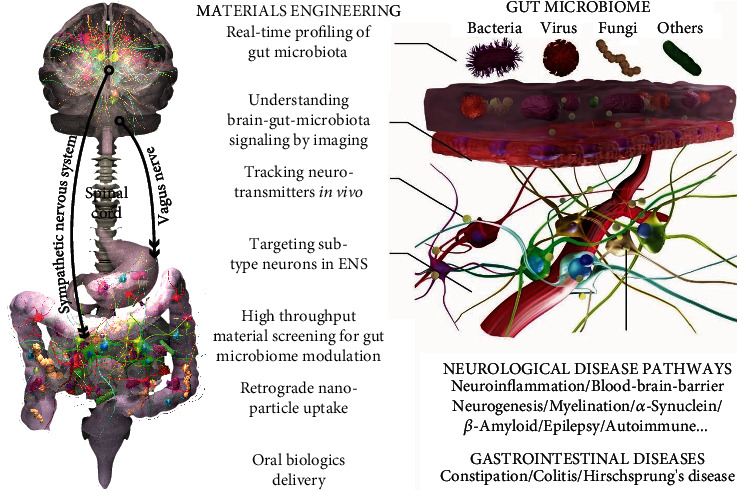
Future directions of biomaterial-based approaches for modulation of gut microbiome and microbiota-gut-brain (MGB) axis.

Diverse neurological disease pathways (e.g., neuroinflammation, neurogenesis, myelination, and protein aggregations) and gastrointestinal diseases (e.g., constipation, colitis, and Hirschsprung's disease) have been associated with the MGB axis [271]. Single-cell omics have also been constantly increasing the complexity of the ENS [272]. Future application of spatial transcriptomics into the analysis of MGB is also expected to offer ever-increasing biological targets for drug development and delivery, opening numerous opportunities for biomaterial innovation (Figure 8). On the one hand, to facilitate these discoveries of novel biological targets, real-time profiling of gut microbiota, *in vivo* tracking of neurotransmitters, and noninvasive imaging of MGB signaling would be tremendously helpful. Advanced sampling methods, biosensors, imaging enhancers, and instrumentation would be the key to realizing these goals. Building more reproducible, more biologically relevant, and more complex *in vitro* models for high-throughput drug screening is another way to accelerate the discoveries of drugs and screening of novel drug delivery devices. On the other hand, how to use these new biological targets to guide the next generation of oral drug delivery systems is equally important. For instance, different subtype neurons play distinctive roles in MGB signaling, but there have been no oral delivery systems that can effectively target subtype neurons. Although advanced viral gene delivery systems have partially realized cell-type-specific delivery into the CNS, their applications in ENS have been limited, especially in the context of oral delivery [273]. *In vivo* screening nanoparticle libraries with diverse charge, size, coating, and targeting ligands and barcoded with DNAs could pave the road for nonviral drug and gene oral delivery to the ENS in a cell-specific manner. In parallel, the spatial heterogeneity of MGB signaling molecules also provides multiple excellent opportunities for developing regiospecific drug delivery devices. Equally importantly, in terms of gut-to-brain signaling, investigating retrograde uptake and transportation of nanoparticles would be of significant interest both for neurological disorder treatment and a fundamental understanding of MGB. Specifically, studies have suggested that protein aggregates such as *α*-synuclein and *β*-amyloid could be mediated by the vagus nerve to the brain and induce Parkinsonism and Alzheimer's disease, but this is under active debate. Nanoparticles that have a similar size to protein aggregates and also advanced imaging modalities may provide excellent insights into these important questions.

### 6.2. Gene-Editing Materials for Modulation of the Gut Microbiome

Control and manipulation of the gut microbiome for therapeutic applications have gained favorable attention due to their significant potential for reducing the incidence and severity of a wide range of human conditions and diseases. However, current approaches, such as antibiotics, broad-spectrum probiotics, and fecal microbiota transplants, nonspecifically perturb the existing microbiome community and potentially impact many species of beneficial commensal bacteria [274]. In this regard, gene-editing tools, which involve the delivery of transgenes into specific members of the endogenous microbiota, have been developed for more specific modulation of the gut microbiome. To date, some gene-editing tools have emerged that have already been implemented, such as transcription activator-like effector nucleases (TALENs), zinc finger nucleases (ZFNs), and CRISPR-Cas systems [275]. These gene-editing techniques enable the addition or removal of the functions of the gut microbiota in a precise way. Specifically, RNA-guided CRISPR-Cas9 systems that have been applied to site-specifically edit bacterial genomes and Ll.LtrB group II introns (retrotransposons that go through RNA intermediary steps) have been successfully repurposed for targeted gene editing of multiple bacteria. Nonetheless, to fully reach their potential and initiate an efficient microbial gene therapy, several advances are needed.

Much research has focused on the bacteriophages-based delivery system to deliver CRISPR-Cas9 for strain-specific depletion and genomic deletions in the gut microbiome [276, 277]. However, the narrow host specificity of the phage limits its application in targeting all pathogenic strains during the gene modification process [277]. To address such an issue and successfully ferry the exogenous plasmids or CRISPR-Cas system to the microbiota, future research should include study of the development of a safe and efficient delivery system for encapsulation and delivery of genome-editing biomacromolecules.

Synthetic materials such as polymers, lipids, and lipid-based nanoparticles have been applied for encapsulating gene-editing tools and delivering them to the cells. For instance, a lipid nanoparticle delivery platform developed by Qiu et al. successfully carries Cas9 messenger RNA and guides RNA for CRISPR-Cas9-based genome editing of Angptl3 *in vivo* [278].

In this regard, we envision that direct, tunable manipulations of the genetic building blocks of microbial communities by deploying synthetic biomaterials will enable novel bioengineering applications in the near future. Also, polymer-based gene delivery systems possess tunable traits such as charge, biodegradability, and molecular weight, thereby yielding enhanced gene-editing efficacy. Synthesized lipid-polymer hybrids have also been reported, and adding lipids to poly-beta amino esters (PBAEs) might improve serum stability and delivery efficiency [278]. However, in *in situ* reprogramming of gut microbiota, there remains to be an arduous and long road.

Material-mediated oral gene modulation tactics are hindered by the degradative conditions of the upper GI tract and poor intestinal absorption [279]. Physiological modification strategies may be needed to suppress gastric acidification to enable a relatively high gene-editing efficiency [280]. Hence, increased knowledge and functional annotation of microbial genomes, coupled with advances in delivery strategy (particularly noninvasive delivery strategies), are required to enhance genome-editing efficiency, to predict the effects of particular genomic modifications, and to maximize the potency of gene-editing tools.

### 6.3. Clinical Translation and Biosafety Regulation

The influences of the gut microbiome on host physiology are so pervasive that the microbiota has been regarded not only as a gripping target for pathogen-related illness but also as an effective prescription to enhance general well-being. Advances in our understanding of the gut microbiome, involving interactions between bacterial species residing in the gut and generation or modification of metabolites that impact host physiology, have brought to light an array of opportunities for the development of novel therapeutics [281].

Accordingly, the potential paybacks of material engineering in the gut microbiome are immense but so are the challenges to reaching these goals. In a recent study, hyaluronic acid-bilirubin-based nanomaterials have been shown to perturb the composition of the gut microbiome in a murine model of acute colitis [254]. Another notable advancement reported by Mosquera et al. revealed that chronic, systemic inflammation arising from a change in the intestinal microbiota sensing in engineered mice reduces the immune response triggered by polymeric nanovaccines [282]. Despite the development of these multifunctional materials for therapeutic modulation of the gut microbiome, translation of laboratory discoveries to the clinic still requires rigorous clinical trials to verify safety and effectiveness. For materials-engineered bacteria, the introduction of exogenous genetic elements in the intestinal microbiota necessitates an understanding of mechanisms that warrant adequate control over the spread of the DNA, along with the bacteria that harbor it [283]. Although nanomaterials can enable the targeted delivery and release of genetic cargoes, horizontal gene transfer may still occur and result in the unintended spread of introduced DNA. Also, effective and safe *in vivo* genome editing needs stringent spatial and temporal control of the genetic cargo activity to diminish off-target mutagenesis [284]. The size of the gene-editing tool, the limitations of delivery vehicles, and the control of nuclease activity should thus be taken into careful consideration. Further, material-mediated oral bacterial therapy requires transportation across physiological barriers, such as the tissue-specific interstitial space and the GI mucus layer. In current clinical trials, the delivery method is mainly based upon oral capsule-mediated delivery [285, 286].

In this context, nanomaterials with therapeutic function and superior protection ability against biochemical barriers (e.g., pH and enzymatic) may play a more important role in future translation. In the next phase of the preclinical study, the considerable variation in the microbiota between individuals should be evaluated, as well as the dynamic ecosystem of human gut microbiota. Given the complexity of the microbiota, manipulating the gut microbiome for therapeutic development requires a more thorough understanding of the precise roles and functions of gut microbiota and their interactions with the host and each other. Such research will likely further emphasize the need for individually tailored nanomedicines. Further, a material-engineered gut microbiome will need to overcome the native microflora's resistance to new residents [286]. Achieving targeted nanomedicines to exert therapeutic functions *in situ* or deliver gene-editing tools for manipulation purposes is of great significance. We envisage that modeling, simulation, and computational analysis can significantly aid the tunable design of nanomedicines, and diverse materials with multiple functions will be inaugurated to hew out the landscape of this field. We also anticipate that the vista of material engineering in the gut microbiome will expand and change rapidly in the near future, yielding novel and safe therapeutics for use by patients and clinicians.
